# Encryption technique based on chaotic neural network space shift and color-theory-induced distortion

**DOI:** 10.1038/s41598-022-14356-x

**Published:** 2022-06-21

**Authors:** Muhammed J. Al-Muhammed, Raed Abu Zitar

**Affiliations:** 1 Faculty of Information Technology, American University of Madaba, Madaba, Jordan; 2 Sorbonne Center of Artificial Intelligence, Sorbonne University-Abu Dhabi, Abu Dhabi, UAE

**Keywords:** Computational science, Computer science, Information technology

## Abstract

Protecting information privacy is likely to promote trust in the digital world and increase its use. This trust may go a long way toward motivating a wider use of networks and the internet, making the vision of the semantic web and Internet of Things a reality. Many encryption techniques that purport to protect information against known attacks are available. However, since the security challenges are ever-growing, devising effective techniques that counter the emerging challenges seems a rational response to these challenges. This paper proffers an encryption technique with a unique computational model that inspires ideas from color theory and chaotic systems. This mix offers a novel computation model with effective operations that (1) highly confuse plaintext and (2) generate key-based enormously complicated codes to hide the resulting ciphertext. Experiments with the prototype implementation showed that the proposed technique is effective (passed rigorous NIST/ENT security tests) and fast.

## Introduction

Information security is an ongoing battle between security experts who strive for devising effective information protection methods and hackers who work persistently to enhance their hacking techniques to breach the information. This battle seems endless and any relaxation from the security experts probably leads to catastrophic surprises. To tip the balance to their favor, security experts have proposed many encryption techniques with different computational models. Traditional techniques use mathematical manipulations and static substitution to encrypt information^1–3^. Biologically based techniques make use of the complexity of human DNA sequences to hide the information^4^. Honey techniques protect the information through deceiving hackers by returning a pleasing, but fake document when attempting the wrong key^5,6^.

Although current standard encryption techniques (e.g. AES) still provide enough protection against current threats, the formidable advancements in cryptanalysis tools may soon challenge these techniques. How the current encryption techniques can effectively face the increasing security challenges or even speculate on the surprises these hacking tools may have is certainly unclear. In such a foggy setting, developing new techniques with more sophisticated computational models sounds logically justifiable. It simply gives more options to respond to emerging security challenges.

This paper offers an encryption technique with an innovative computational model. The technique uses a chaotic space shift scheme to deeply transform plaintext symbols and shift them to a totally different domain that does not correlate with the original. Furthermore, the technique uses operations whose computations are based on principles inspired by the color theory. These operations use the encryption key to generate enormously complicated codes that provide an impenetrable shield in which the ciphertext symbols are hidden. Combining chaotic systems with principles from color theory offers a unique encryption technique with an extremely sophisticated computational model that can effectively protect information against hacking techniques.

The contributions of this paper can be summarized as follows. The paper proposes a space-shift scheme that transforms the input blocks to ciphertext with high confusion and diffusion. Since the space-shift scheme is fundamentally founded on chaotic substitution and distortion operations, the confusion induced by the proposed scheme largely outperforms that induced by current methods that rely on static substitution and symbol manipulation. The paper also proposes a computationally-light method inspired by the color theory principles. This method conservatively uses the key to generate enormously complicated codes for sealing the ciphertext symbols.

We present our contributions as follows.  "Chaotic system: Lorenz chaotic system" Section introduces the chaotic system and gives the details of o novel way for initializing the chaotic system parameters. Sections Space chaotic shift process and Space chaotic shift process inverse discuss the space chaotic shift and its inverse. “Section 5” presents the technical details of the color-theory and key based process for generating sealing codes. “Section Encryption/decryption process” presents the encryption and decryption technique. We analyze the performance of the proposed technique in “Section Performance analysis” and discuss the performance analysis results in “Section Discussion”. We compare the proposed technique to state-of-the-art techniques in “Section 9” and give concluding remarks and directions for future work in “Section Conclusions and future work”.

## Chaotic system: Lorenz chaotic system

The Lorenz is a hyperchaos system with non-periodic behavior due to its high sensitivity to the initial conditions^7–9^. This property is ideal for cryptography techniques because it enables the generation of long sequences of chaotic values without repeated patterns and any changes to the initial conditions results in very different sequences. Lorenz system is defined by the differential equations (1)^10–13^.

1 $$\begin{aligned} \begin{aligned}{}&f_x(x, y, z) = \frac{dx}{dt} = a(y - x) \\&f_y(x, y, z) = \frac{dy}{dt} = cx-y-xz \\&f_z(x, y, z) = \frac{dz}{dt} = xy - bz \end{aligned} \end{aligned}$$

The values *a*, *b*, and *c* are the parameters of Lorenz system. As reported in^14^, Lorenz system falls into a hyperchaotic state for *a* = 10, *b* = 8/3, and *c* = 28. The effective intervals for the initial variables $x_0$, $y_0$, and $z_0$ are: $x_0$, $y_0 \in$ (−40, 40) and $z_0 \in$ (1, 80). To use Lorenz chaotic system, it must be discretized. Although many discretization methods, we use the fourth-order Runge-Kutta method, which can be found in^14^.

The effective use of Lorenz system in cryptography requires binding the parameters ($x_0$, $y_0$, $z_0$, *a*, *b*, *c*) to values computed using the key. To the best of our knowledge, all the encryption techniques that use Lorenz system require users to provide additional values to initialize these parameters. One different way to bind Lorenz parameters is to create a seed from the key and use it in one of the known random number generators to initialize the parameters. This way is not cryptographically sound. First, the seeds for a random generator can hold far fewer bits than available in the key, which means that the generator does not fully exploit the key. Second, random number generators are not generally cryptography secure. This paper proposes an innovative technique that fully exploit the key to automatically bind Lorenz system’s parameters.

### Lorenz system initializer

**Figure 1:**
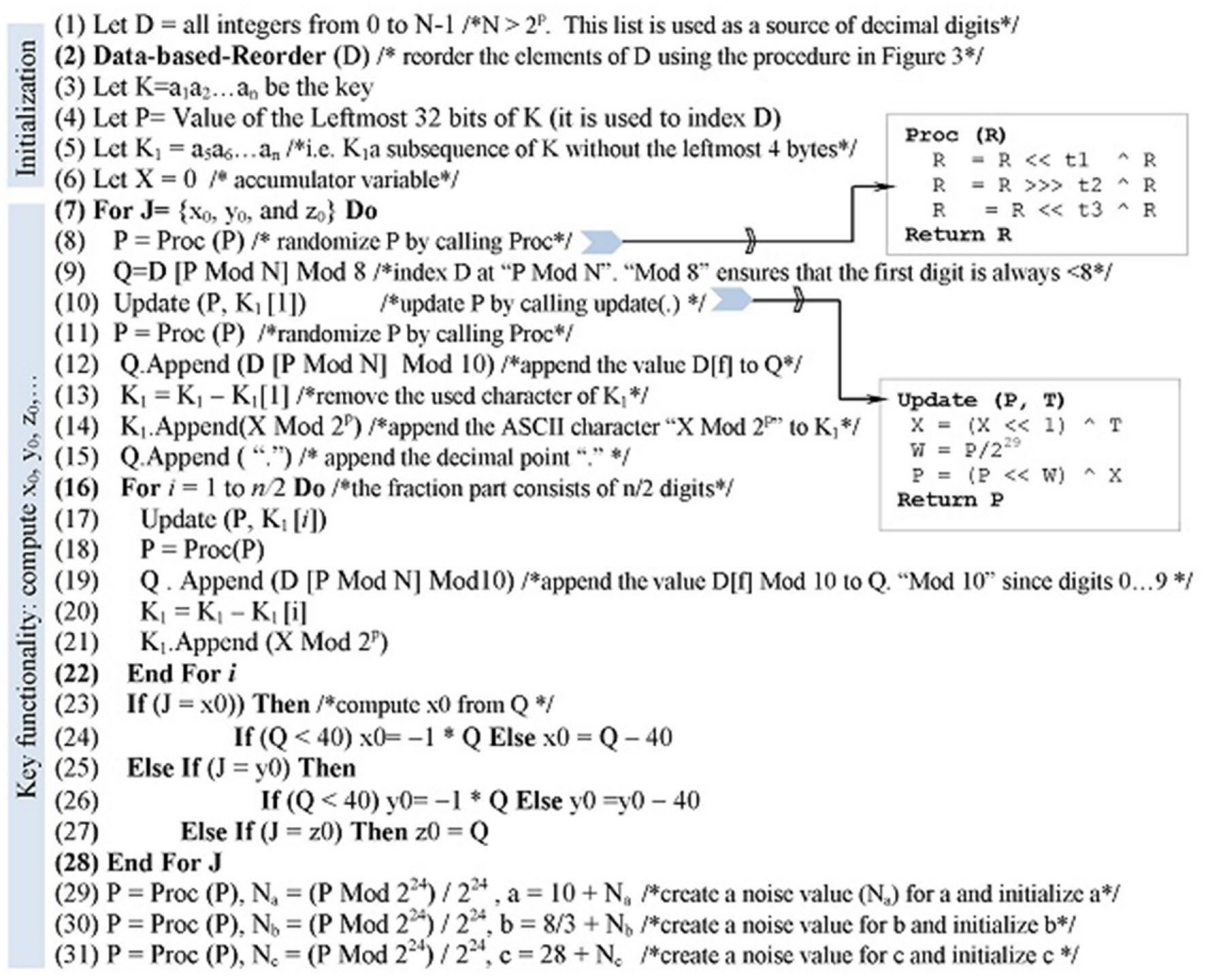
Process for producing initial values for Lorenz chaotic system.

The initializer is an efficient technique that securely exploits all the bits of the key. Figure 1 delineates the initialization process. The proposed process computes values for Lorenz’s parameters using the key and it extremely sensitive to the key variations—a single bit change in the key causes drastic changes to the computed values.

**Figure 2:**
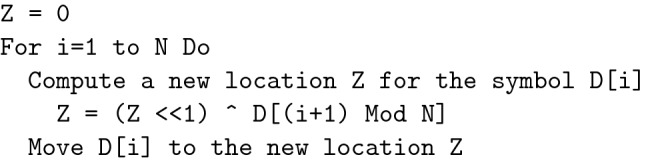
Data-dependent reordering.

Although the process is well-documented (see Fig. 1), we briefly describe its core functionality. The input to the process is the *n*-symbol key *K*, where each symbol is *p* bits. The output is three values for the parameters of Lorenz chaotic system ($x_0, y_0, z_0$) and three random noises for distorting the system coefficients (*a*, *b*, *c*). The process uses the variables *D*, *P*, *X*,  and $K_1$, which are initialized as specified. The list *D* is populated with the integers from 0 to *N*–1 ($N > 2^p$) and these integers are scattered using the operation Data-based-Reorder (*D*) (Fig. 2). The variable *X* combines all the processed symbols of the key so that a change in any key symbol necessarily causes changes to the all subsequent uses of *X*. The variable *P* is initialized with the leftmost 4 bytes of the key *K*. The variable $K_1$ is assigned the rest of the key’s bytes and its value is constantly updated at each iteration by appending the ASCII character “$X~Mod~2^p$ ” to it.

The loop (7 through 26) binds the parameters of Lorenz system ($x_0, y_0, z_0$) with appropriate values. Each iteration of the loop involves randomizing *P* by calling *Proc*(*P*) operation, updating *P* by calling *update*(.) operation, and indexing *D* using “*P* *Mod* *N*” to retrieve an integer and append it to *Q*. The local variable *Q* can be $x_0, y_0$, and $z_0$. The operation *Proc*(.) randomizes its input using an XOR operation ($\wedge$) and a series of logical shifts ($<<$ or $>>>$). (Authors^15^ provided many possible assignment values for the shift amounts $t_1, t_2$, and $t_3$—e.g. 35, 21, and 4.) The operation *update* (.) updates *X* using the input symbol *T* and then uses updated *X* to update *P* by left shifting *P* by *W* and then XORing the outcome with *X* (*W* is the leftmost 3 bits of original *P*—before refreshing its value).

Step 9 ensures that the leftmost digit of $x_0, y_0$, and $z_0$ is less than 8. That is because the rang of these three variables is less than 80. Since $x_0$ and $y_0$ $\in$ (−40, 40), the initializer interprets the value of Q as follows. For $x_0$ and $y_0$, if $Q <40$ assign $-1*Q$ to $x_0$ or $y_0$ else assign $Q-40$. Since $z_0 \in$ (1, 80), the value of *Q* is directly assigned to $z_0$ without further processing.

The instructions 27–29 compute noise values $N_x \in [0, 1]$ for the parameters of Lorenz system (*a*, *b*, *c*). Each noise $N_x$ is computed by updating the variable *P* and dividing the value of the rightmost 24 bits by the maximum value of 24 bits (i.e. $2^{24}$).

### Chaotic number generation

After initializing its parameters, the Lorenz system is used to produce three random streams of numbers corresponding to the three parameters *x*, *y*, and *z*. For a maximum effectiveness of the generated streams, the system is iterated for *H* times to ensure true transition to the chaotic state. After these *H* iterations, the streams ($X_i, Y_i, Z_i$) are generated using formulas (2). Where *floor*(*x*) returns the closet integer to *x* and *k* represents the desired range of chaotic numbers (e.g. if the desired range is the integers 0...255, then *k* =256).

2 $$\begin{aligned} \begin{aligned} {}&X_i = floor (x_i \times 10^{14})~ MOD~ {k}\\&Y_i = floor (y_i \times 10^{14}) ~MOD~ {k}\\&Z_i = floor (z_i \times 10^{14}) ~MOD ~{k} \end{aligned} \end{aligned}$$

## Space chaotic shift process

**Figure 3:**
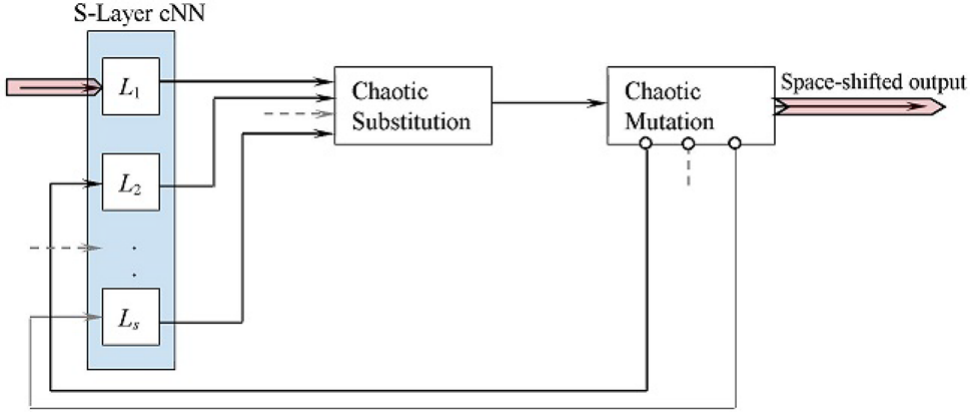
The space chaotic shift process: flow of control.

Figure 3 shows the flow of control of the space chaotic shift process. It handles a plaintext block by repeatedly executing (*s* times) the subprocesses S-Layer cNN, Chaotic Substitution, and Chaotic Mutation. Each execution of these subprocesses enormously shifts the input symbols from the plaintext space to a completely different space. As such, the process introduces maximum confusion to the encryption technique.

### Finite Galois field GF($2^p$)

The Galois field, denoted GF($$2^p)$$, contains a finite number of elements, where these elements are integers mod *p*. We succinctly discuss some of the Galois field GF($$2^p)$$ results and invite respected readers to see^16,17^ for detailed discussion.

Each element $$i \in$$ GF($$2^p)$$ is represented as an irreducible polynomial. For instance, the representation of the decimal value 177 (10110001) is $$x^7+x^5 + x^4+1$$. The four arithmetic operations addition, subtraction, multiplication, and division are different from their intuitive meaning. The addition “+” and subtraction “–” of two elements $$i_1, i_2 \in GF(2^p)$$ are performed as an XOR operation between these two elements ($$i_1 ~^{\wedge }~ i_2$$). The multiplication ($$\otimes$$) and division (/) are rather tricky. The multiplication of two elements is defined as a polynomial multiplication for these two numbers modulo an irreducible polynomial in GF($$2^p$$). The division is defined likewise except that the first element is multiplied by the multiplicative inverse of the second element. We illustrate the multiplication using an example from^17^—3*F* and *A*5 are two hexadecimal numbers.

$$\begin{aligned} \begin{array}{lll} {3F\odot A5} &{} = &{} (00111111)_2 \otimes (10100101)_2 \\ {} &{} = &{} (x^5+x^4+x^3+x^2+x+1)\otimes (x^7+x^5+x^2+1) \\ {} &{}= &{} x^{12}+x^{11}+2x^{10}+2x^9+2x^8+3x^7+2x^6+3x^5+2x^4+2x^3+2x^2+x+1 \\ {} &{} = &{} x^{12}+x^{11}+x^7+x^5+x+1 \\ {} &{} = &{} 1100010100011_2 \end{array} \end{aligned}$$

Observe, since addition in GF$$(2^p)$$ is an XOR, the terms with even coefficients are eliminated. To find the final result, we must mod the result ($$1100010100011_2$$) by the irreducible polynomial ($$100011011_2)$$, which can be done by long division with XOR ($$^{\wedge }$$) in place of subtraction as follows.

$$\begin{aligned} \begin{array}{lll} {1100010100011_2 \,\,mod\,\, 100011011_2} &{} &{} \\ {} &{} = &{} (1100010100011)~^{\wedge }~(100011011) \\ {} &{}= &{} (100100010011)~^{\wedge }~(100011011) \\ {} &{} = &{} (111001011)~^{\wedge }~(100011011) \\ {} &{} = &{} 11010000=D0~(the~result~of~the~ multiplication)\end{array} \end{aligned}$$

### Chaotic neural network subprocess (*S*-Layer *cNN*)

The *S*-Layer *cNN* subprocess consists of a network of *s* layers and a processing logic that uses operations on Galois field. The *s* layers are chained in Hill cipher manner, where the output of the layer $L_i$ is the input for the next layer $L_{i+1}$ (after receiving additional processing). Each layer $L_i$ is an $n \times n$ array whose entries are chaotic values from Galois field GF($2^p$) produced as follows. Let ($x_j, y_j, z_j$) be triples of chaotic numbers generated by the chaotic system. We compute new chaotic values $w_j$ = *MOD* (*floor*$\langle$($x_j+y_j +z_j$) * $10^{14}$ $\rangle$, $2^p$) and assign them to $L_i$. Where *floor* (*t*) returns the closest integer greater than or equal to *t* and *MOD* is modulo operation. Note because of the module by $2^p$, $w_j$ are values in GF(2$^p$).

The *cNN* subprocess manipulates an *n*-symbol input block *Q* (which is interpreted as a vector of *n* entries) and produces a new *n*-symbol block *R* using the following matrix multiplication *R* = $L_k \bigotimes Q$ (or $r_i$=$\sum _{j=1}^{n} l_{i,j}\bigotimes q_j$, *i*=1...*n*). The chaotic numbers $l_{i,j} \in L_k$ and the characters $q_j$ of the input block *Q* are to be treated as values in the field GF(2$^p$). The sum ($\sum {}{}$) and the multiplication ($\bigotimes$) are operations on the field GF(2$^p$)—not classical sum and multiplication.

The processing logic for *cNN* subprocess is reversible. To restore the original block *Q* from *R* = $L_i \bigotimes Q$, the inverse of $L_i$ is computed using the operations of GF(2$^p$). The inverse can be computed using any of the Matrix algebra techniques. By computing the inverse matrix $L_i^{-1}$, *Q* can successfully be recovered by *Q*= $L_i^{-1} \bigotimes R$.

**Figure 4:**
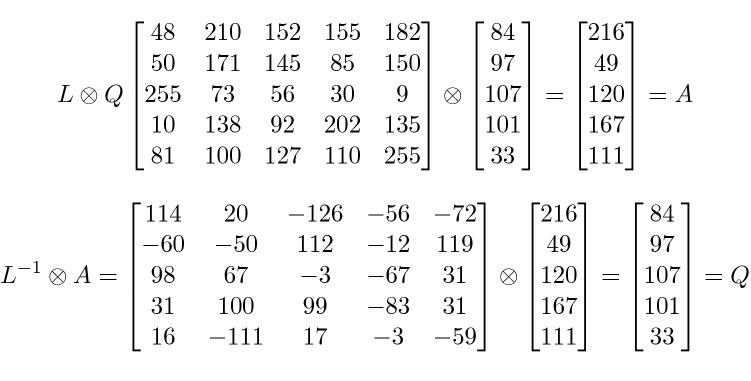
An example of the chaotic neural network computation.

Figure 4 shows an example of the computation of the chaotic neural network. The top part of Fig. 4 shows the result *A*(216, 49, 120, 167, 111) of multiplying the chaotic matrix *L* with the input vector *Q*(84, 97, 107, 101, 33). Each element of *A* is computed by multiplying a row of the matrix *L* with the vector *Q*. For instance, 216 = 48 $\otimes$ 84 + 210 $\otimes$ 97 + 152 $\otimes$ 107 + 155 $\otimes$ 101 + 182 $\otimes$ 33, where $\otimes$ and + are the multiplication and addition under GF($2^p$) and are performed as described in “Section [Sec Sec6]”. The bottom part of Fig. 4 shows that the computation is reversible using the inverse of the matrix $L^{-1}$.

### Chaotic substitution subprocess

**Figure 5:**
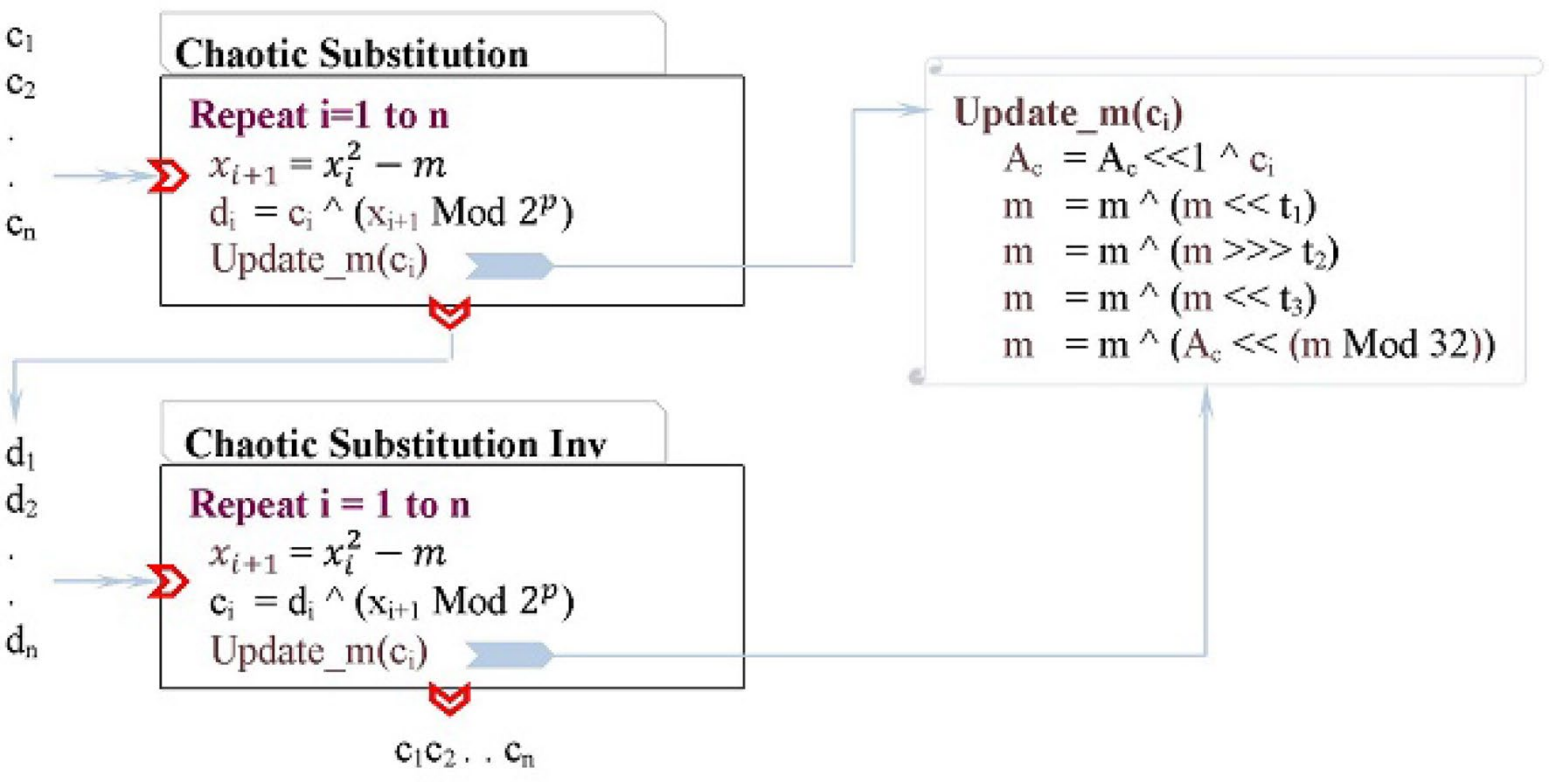
The chaotic substitution (top) and the chaotic substitution inverse (bottom).

The chaotic substitution subprocess is defined in Fig. 5. The chaotic behavior of the substitution subprocess is stimulated by Eq. (3), where $m\in [1, \infty$) and $x_i$ is a long positive integer. Equation (3) resembles the effective one-dimensional logistic chaotic map but with a better correlation to the input symbols due to the way in which the parameter *m* is updated. As Fig. 5 shows, the operation Update_m($c_i$) updates *m* using a input-dependent process. First, the processed input symbols $c_1c_2...c_{i-1}$ are accumulated by the variable $A_c$ (initially zero) through Shift/XOR operations. The variable $A_c$ is therefore a “memory” that remembers the impact of the previously processed symbols and passes this impact to influence the processing of the current input symbol $c_i$. Second, the parameter *m* is randomized using a series of Shift/XOR operations that ensure deep bit-mixing. Third, *m* is updated by combining its previous value with the impact of the effect-carrying memory $A_c$ (the combination is performed via Shift/XOR operations). With this update to *m*, the computational behavior of Eq. (3) is very sensitive to the input and chaotic.

3 $$\begin{aligned} x_{i+1} = (x_i^2 - m) \end{aligned}$$

The chaotic substitution substitutes the block $c_1c_2...c_n$ using Eq. (3) as outlined in Figure 5 (left-top box). The parameters *m* and $x_i$ of the Eq. (3) are initialized to two chaotic values obtained from the chaotic system. The input symbol $c_1$ is substituted with the symbol $d_1$, which is computed using the initial values of $x_i$ and *m* since we do not have any previous input symbols. The input symbols $c_i$ ($i>1$) are substituted with the symbols $d_i$ that are computed using the updated values of $x_i$ and *m*. As such, the substitution of every input symbol $c_j$ is influenced by the preceding input symbols $c_k$ (*k*=1...$j-1$).

The chaotic substitution subprocess is reversible provided that *m* and *x* are initialized to the same values. (The chaotic generator guarantees initializing *m* and *x* to the same values—used during the substitution—provided that the correct encryption key is provided.) Figure 5 (left-bottom box) shows the algorithmic steps of Chaotic Substitution subprocess Inverse, which effectively restores the plaintext block from the substituted block. The inverse subprocess essentially performs the same steps as the Chaotic Substitution subprocess except that the input is the substituted block.

### Chaotic mutation subprocess

This subprocess increases the confusion of the block manipulation process. The movement of a crawler within a two-dimensional space (called mesh) may invoke a destructive behavior in the mutation subprocess that causes deep changes to the input symbols. We describe first the mesh and then show how the crawler’s movement may stimulate the mutation subprocess’s destructive behavior.

#### The mesh

**Figure 6:**
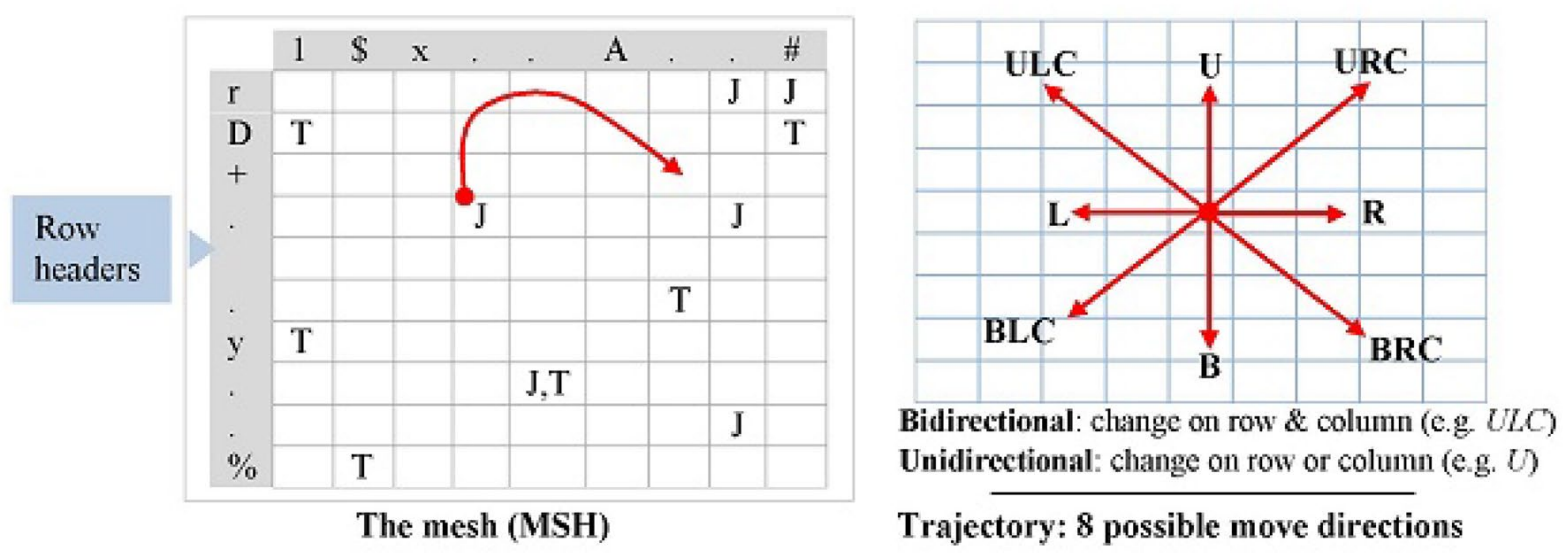
The mesh MSH (left) and eight instances of trajectory (right).

The mesh is an $N\times N$ space, denoted by *MSH*. Figure 6 (left) provides an example of the mesh. The headers of the columns and the rows are labeled with ASCII codes from 0 to $N-1$. ($N=2^p$; *p* is the number of bits that represent the used symbols.) The codes of the columns/rows are randomly scattered by swapping the content of the entries at indices *i* and $z_i$, where $z_i$ is a chaotic value. A number $M = N^2/2\times (1+t_v/2^p)$ of the mesh’s entries are designated with the directives T and J, where $t_v$ is a chaotic value less than or equal to $2^p$. The count of the T and *J* is specified as follows: *M*/2 of the Mesh’s entries are annotated with T and *M*/2 of the entries are annotated with J. *M*/2 cells of the mesh are annotated with the directive T using *M*/2 chaotic indexes ($i_1, i_2$). *M*/2 cells of the mesh are annotated with the directive J likewise. It is likely that some of the entries are annotated by both T and J due to the chaotic annotation. Along with the annotated mesh, we envisage a crawler that starts from an initial point (*x*, *y*) within the mesh and moves over the mesh’s cells according to logic to be made precise later.(As we discuss next, the directive T triggers the mutation process while the directive J fuzzifies the crawler’s move within the mesh.)

Figure 6 (right) also shows **Trajectory**—a variable determines a move direction. The Trajectory defines 8 direction flags, which constitute all the possible moves within the mesh starting from some cell. Half of the direction flags are bidirectional because they entail changes on the rows and the columns. The flag URC is an example because a move along this flag causes the value of the rows’ index to decrease and the value columns’ index to increase. The other half of the direction flags are unidirectional because they entail change on either the rows or columns. The flag B is an example because moving along this flag causes the value of the row’s index to increase.

#### Mesh-based chaotic mutation

**Figure 7:**
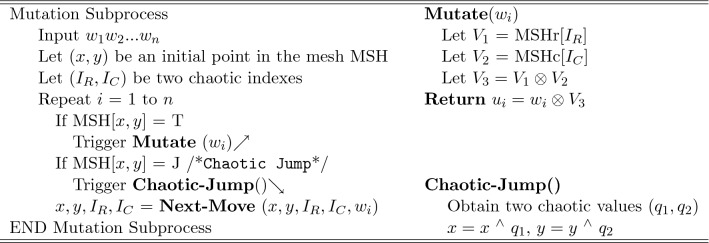
The mutation subprocess.

The mutation subprocess processes the input block $$w_1w_2...w_n$$ using the algorithmic steps in Fig. 7. First, the crawler’s initial position (*x*, *y*) and the variables ($$I_R, I_C)$$ are initialized with chaotic values. When the mutation subprocess considers an input symbol $$w_i$$, it triggers the *Mutate*(.) operation only if the crawler’s current position (*x*, *y*) is annotated with T (i.e. if MSH[*x*, *y*] =T). When invoked, *Mutate*(.) uses the variable $$I_R$$ to index the row’s headers (denoted by $$MSH_r[I_R]$$) to obtain a symbol $$V_1$$ and uses the variable $$I_C$$ to index the column’s headers (denoted by $$MSH_c[I_C]$$) to obtain a symbol $$V_2$$. It uses then the values $$V_1$$ and $$V_2$$ to mutate the input symbol $$w_i$$ using Galois multiplication ($$\otimes$$) operation as illustrated in the figure.

**Figure 8:**
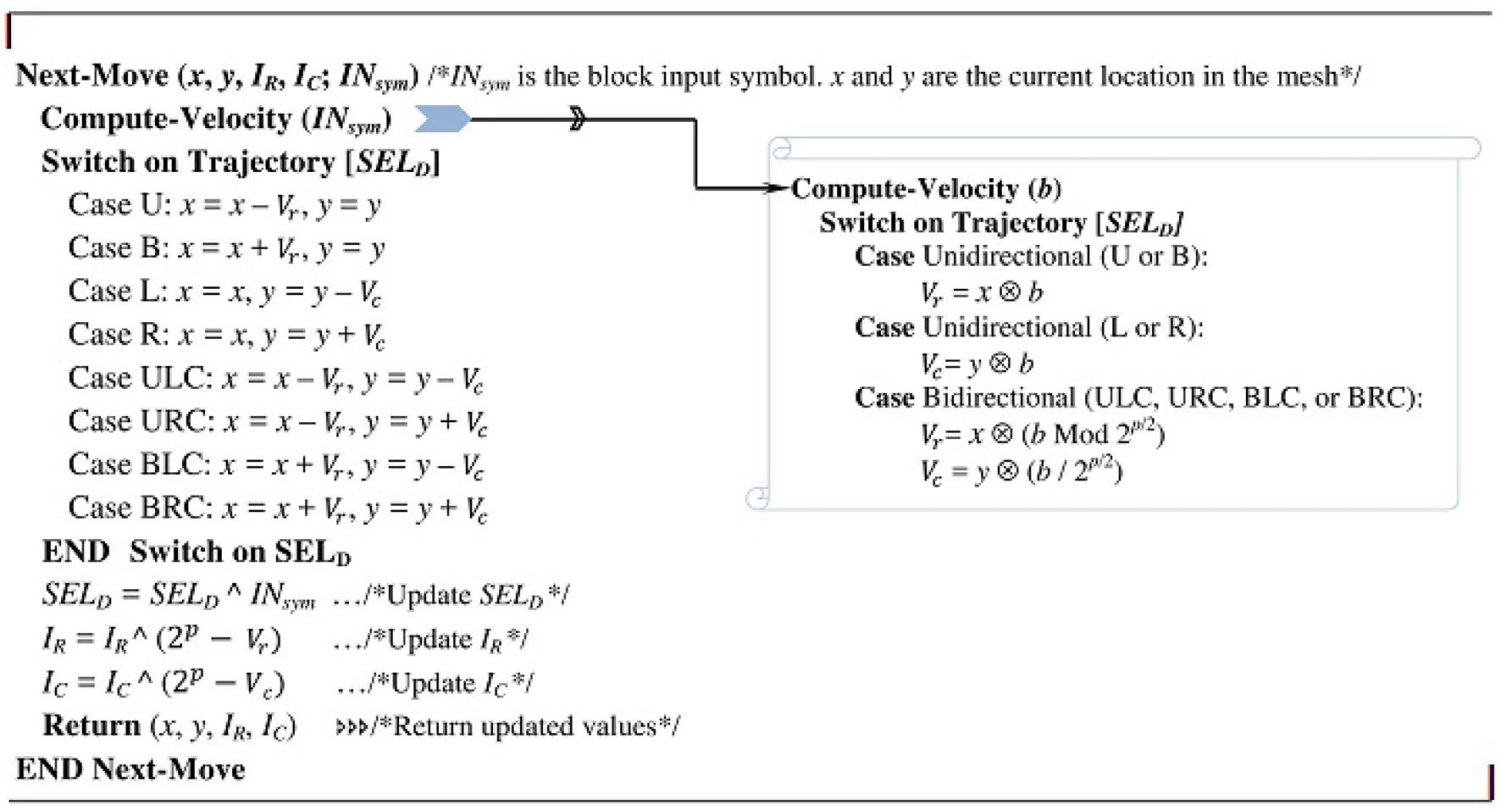
Computing the next move parameters: algorithmic instructions.

The mutation subprocess updates the crawler’s position by executing the operations Chaotic-Jump() and Next-Move(). The operation Chaotic-Jump() is conditionally executed: if the current cell of the mesh (MSH[*x*, *y*]) is annotated with J. If executed, Chaotic-Jump() uses chaotic values to distort the crawler’s current position. It does so by XORing the current position (*x*, *y*) with a pair of chaotic values ($q_1, q_2$) obtained from the chaotic system. The Next-Move() operation whose algorithmic steps are defined in Fig. 8 imposes an input-dependent update to both the crawler’s current position (*x*, *y*) and to the variables ($I_R$, $I_C$) regardless whether MSH[*x*, *y*] is annotated with J. Referring to Fig. 8, Next-Move() executes the routine Compute-Velocity() to produce the velocity $V_r$ or $V_c$ or both depending on the value of Trajectory[$SEL_D$]. ($SEL_D$ is initialized to a chaotic value and updated as shown in Fig. 8.) When Trajectory[$SEL_D$] is unidirectional and equals U or B, the routine computes $V_r$ by performing Galois multiplication ($\otimes$) between the input symbol *b* and the coordinate *x*. When Trajectory[$SEL_D$] is unidirectional and equals L or R, the routine computes $V_c$ by performing Galois multiplication ($\otimes$) between the input symbol *b* and the coordinate *y*. When Trajectory[$SEL_D$] is bidirectional however, the routine computes $V_r$ and $V_c$ by respectively multiplying ($\otimes$) the coordinate *x* with the value of the right half bits of *b* (i.e. by *b* Mod $2^{p/2}$) and multiplying the coordinate *y* by the value of the left half bits of *b* (i.e. by *b* / $2^{p/2}$).

The Next-Move(.) operation uses the computed velocities $V_r$ and $V_c$ to update the value of either *x* or *y* or both based also on Trajectory[$SEL_D$]. When the Trajectory[$SEL_D$] is a unidirectional flag (U,B, L, or R) only *x* or *y* is updated. For instance, if Trajectory[$SEL_D$] = U (direction of the move is “up”) only *x* is updated by subtracting the velocity $V_r$; no changes to *y* because the move is along the same column. When Trajectory[$SEL_D$] is bidirectional (ULC, BLC, URC, or BRC), both *x* and *y* are updated. For instance, if Trajectory[$SEL_D$] = ULC (direction of the move is “upper left corner”), *x* and *y* are updated by subtracting the velocity $V_r$ from *x* and the velocity $V_c$ from *y*. The velocities $V_r$ and $V_c$ are also used to update chaotic indices $I_R$ and $I_C$ through an XOR operation as illustrated in Fig. 8.

According to the Figs. 7 and 8, the Mutation subprocess is data and encryption key dependent. The input symbols influence the move of the crawler within the mesh because these symbols are used to calculate the velocities and to update the variable $SEL_D$, which affects how the current position of the crawler is updated. The encryption key also controls the move of the crawler. The Chaotic-Jump() operation shoots the crawler’s current position within the mesh using chaotic values generated based on the key. The key determines the number of the directives T and J and their topology (distribution) within the mesh. Each key impacts the number of the directives J and T and their topology within the mesh differently. More T-annotations cause more input symbols to be processed and more J-annotations cause more chaotic changes to crawler’s position, largely affecting which symbols to be processed. The topology of T and J also determines which symbols in the input block to be processed. This dual-dependency of the mutation subprocess on the input and key makes it so effective in inducing a great confusion in the output.

**Figure 9:**
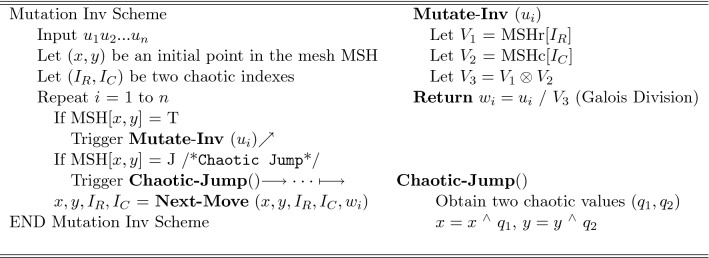
The mutation inverse scheme.

The mutation subprocess is reversible. Figure 9 delineates the inverse steps. Note, the inverse mutation subprocess executes roughly the same steps as the mutation subprocess. The key difference is that in the mutation subprocess inverse, the input symbol $u_i$ is divided by $V_3$ instead of multiplied.

## Space chaotic shift process inverse

This process reverses the impact of the space chaotic shift process and restores the plaintext block. The inverse process uses pretty similar processing steps as the space chaotic shift process (Fig. 3) with some modifications. First, the inverse process uses the inverse of each subprocess. Second, the inverse process executes the inverse subprocesses backwards. Therefore, if we denote inverse of the chaotic mutation subprocess by $C^{-M}$, the inverse of the chaotic substitution subprocess by $C^{-S}$ and the inverse of the chaotic neural network subprocess layer $L_i$ by $L_i^{-1}$, the sequence of the execution would be $\prec C^{-M}\mapsto C^{-S} \mapsto L_s^{-1} \succ ~ \mapsto \cdot \cdot \cdot \mapsto ~ \prec C^{-M}\mapsto C^{-S} \mapsto L_1^{-1}\succ$.

## Sealing layer

**Figure 10:**
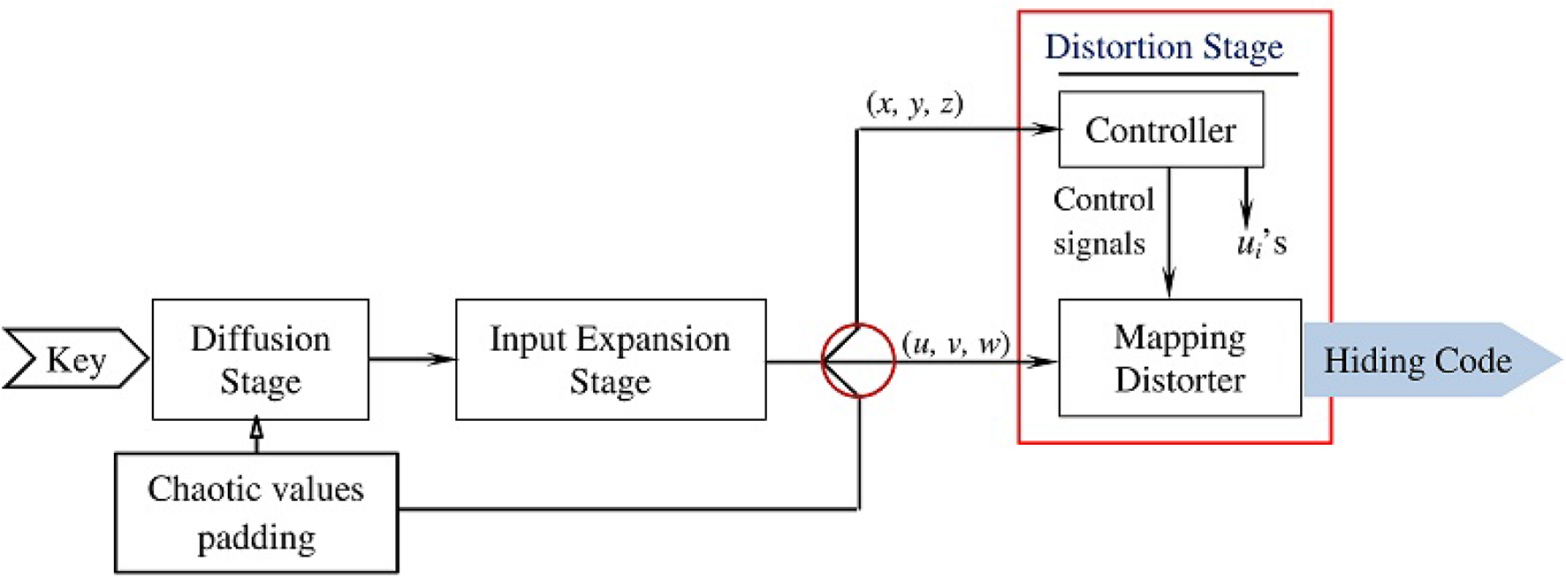
The sealing layer stages.

The sealing layer generates key-based enormously complicated codes that seal the ciphertext symbols and greatly confuse the ciphertext (the output of the space shift stage). Figure 10 shows the core components of this layer and the flow of control. The sealing layer receives the encryption key, which is processed by the Diffusion stage. The Input Expansion stage expands the diffused key to a sequence of *d* bytes. The Distortion stage uses the left $d_1$ bytes of the output sequence and generates hiding codes. The right $d_2$ bytes are fed back to the diffusion stage for producing more expanded sequences. To impose more confusion on the input of the Diffusion stage, the $d_2$ bytes feedback are noised by padding chaotic values to it (obtained from stream *Z* of the chaotic generator). The number of padded chaotic values can be between 0 and $d_2$ (the length of the feedback) and this number is determined by the first chaotic number to be padded as follows. If *H* is the first chaotic value, the number of chaotic values to be padded to the feedback is “*H* Mod $d_2$”. This chaotic noise is extremely important because it greatly varies the input to the diffuser, which complicates the generated key-based sealing codes. The following subsections present the technical details of the sealing layer stages.

### Diffusion stage

**Figure 11:**
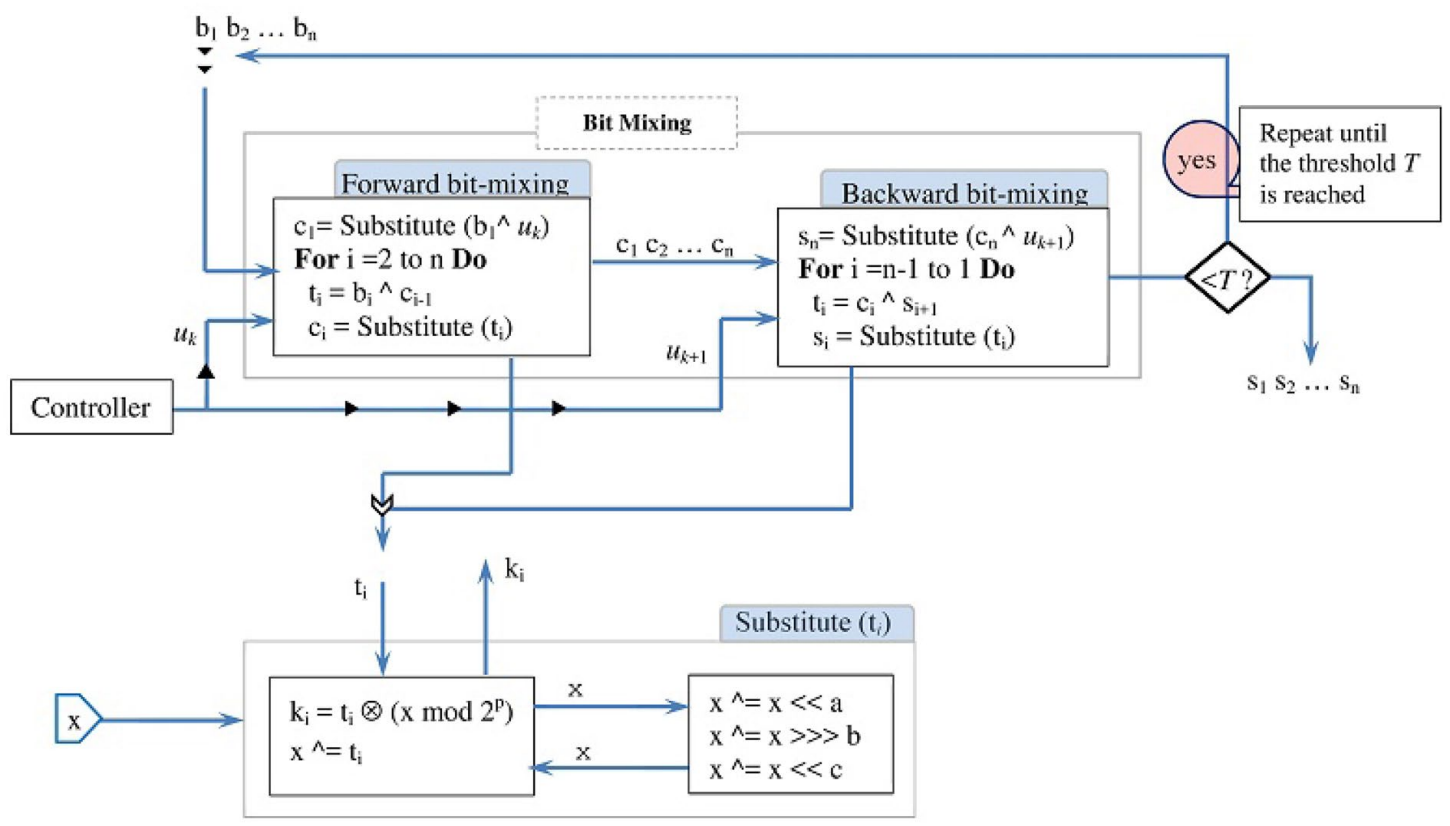
The diffusion stage processing logic.

The diffusion stage processes its input in many rounds as illustrated in Fig. 11. Each round executes forward and backward bit-mixing along with a chaotic substitution.

The forward bit-mixing handles the input $b_1b_2...b_n$ using substitution and XOR instructions. The symbol $b_1$ is handled by XORing (“$^\wedge$”) it with a value $u_k$ provided by the controller and the outcome of the XOR operation is substituted using the Substitute() operation to yield $c_1$. The symbols $b_i$ ($i>$1) are handled by XORing each symbol $b_i$ with the previous symbol $c_{i-1}$ and then substituting the outcome of the XOR operation. The backward bit-mixing uses similar logic except that it starts from the end of the input block $c_1c_2...c_n$. It handles the symbol $c_n$ by XORing it with a value $u_{k+1}$ provided also by the controller and substitutes the outcome. The remaining symbols are manipulated using similar logic as the forward bit-mixing.

The Substitute() operation substitutes its input argument $t_i$ using a stochastic behavior. This operation uses a 64-bit variable *x* (initialized with a chaotic value) to accumulate the impact of the previously processed input symbols through an XOR operation ($x~^\wedge$=$t_i$). The stochastic behavior is induced by a system of XOR-Shift operations that randomize the variable *x*. (The values of *a*, *b*, and *c* are specified as in^15^.) The Substitute() operation processes its input argument $t_i$ by first randomizing the variable *x* using the system of XOR-Shift and then multiplying $t_i$ by “*x* mod $2^p$”. Respected readers should interpret “$\otimes$” as the multiplication in Galois field GF($2^p$). Additionally we take “mod $2^p$” since each input symbol consists of only *p* bits.

The diffusion stage is the primary source of confusion for the sealing layer. The bidirectional bit-mixing enables the diffusion stage to detect the change wherever occurs in the input and transmit this change back and forth to cover all the output symbols, causing every output symbol to change. The stochastic substitution operation props the sensitivity of the bit-mixing. Because the accumulator variable *x* collects the impact of all previously processed input symbols, any change in the previously processed symbols impacts the substitution of the subsequent input symbols. This props the sensitivity of the forward/backward bit-mixing due to embedding variations of the input in the processed symbols.

### Input expansion stage

The sealing code generation requires continuous expansion of the key to produce arbitrary length hiding codes. Although literature has reported many techniques that can expand a sequence of *n* symbols to a longer sequence (e.g.^18,19^), this paper utilizes SHA-512^20^. SHA-512 is a one-way, fast, and has high avalanche effect; making it a perfect choice for key expansion. It hashes a string *S* with a size of $\lambda$ bits ($0 \le \lambda \le 2^{128}$) and produces a corresponding hash value of length 64 bytes (512 bits). (The technical details of SHA-512 is beyond the scope of the paper and interested readers are kindly referred to^21^.) We use the leftmost 48 bytes as an input to the Distortion stage and the right 16 bytes as a feedback to Diffusion stage for producing more sequences.

### Distortion stage

The distortion stage uses an effective computational model whose functionality is based on physical principles inspired by the color theory. In particular, the distortion stage uses computations that are based on the color theory to generate distortion values and trigger distortion operations that consume the generated distortion values to deeply distort the input symbols. As Fig. 10 shows, the distortion stage continuously consumes two triples (*x*, *y*, *z*) and (*u*, *v*, *w*) and produces an output triple (sealing code).

#### The controller

**Figure 12:**
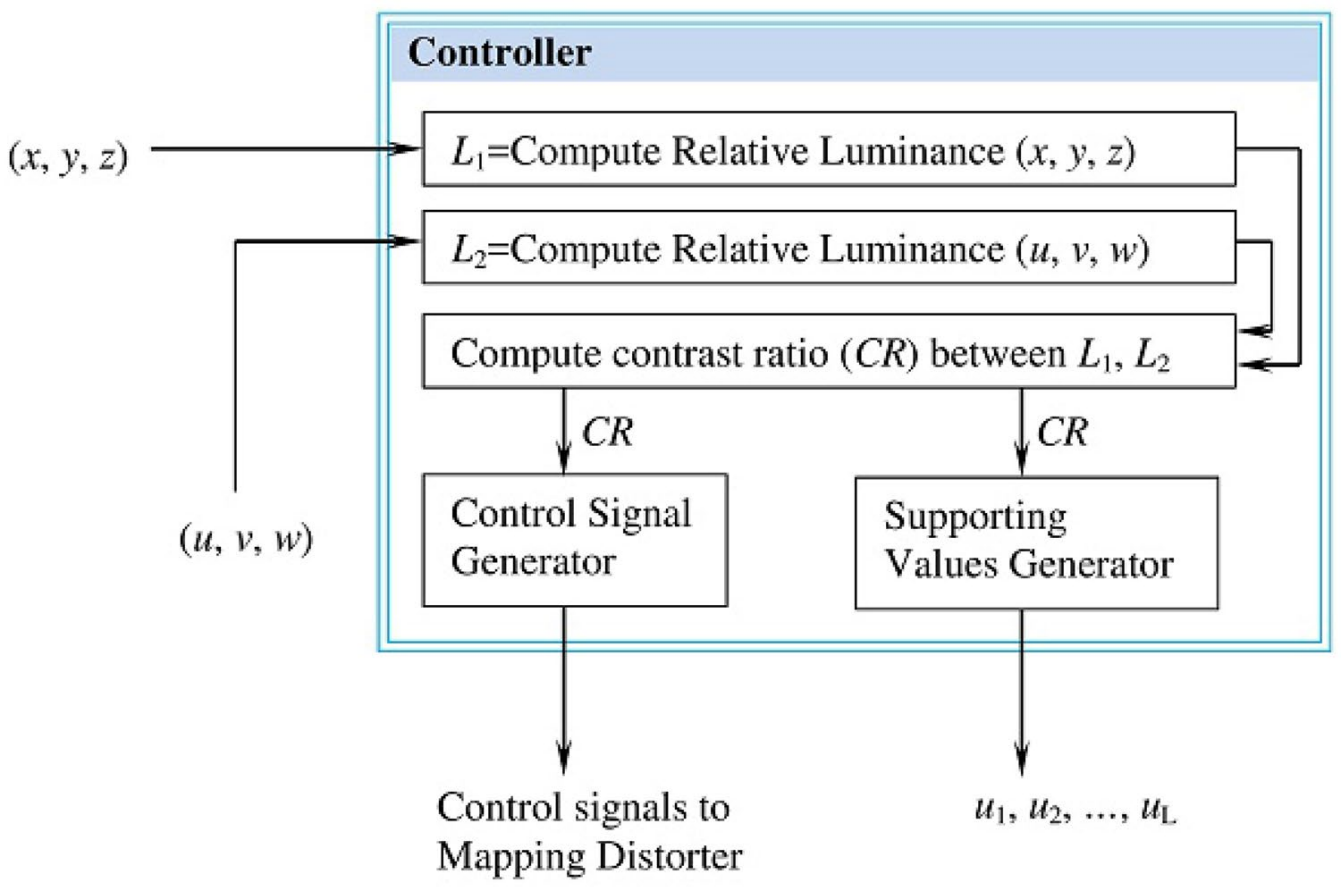
The controller logic.

Figure 12 outlines the inputs and the outputs of the controller. The controller generates four control signals (Map, Map-Distort, Distort-Map, and Skip) that adjust the functionality of the mapping distorter—i.e. determine how the mapping distorter processes its input triples. The four control signals are described in Table 1. The controller generates these four signals using two fundamental themes inspired from the Color theory: *relative luminance* and *color contrast ratio*^22,23^. Given an input triple (*x*, *y*, *z*), which can be interpreted as a triple of color code RGB (red, green, blue), the controller computes the relative luminance for this triple using Eq. (4)^23^.

4 $$\begin{aligned} L = 0.2126 * R + 0.7152 * G + 0.0722 * B \end{aligned}$$

Where, the linear values R (red), G (green), and B (blue) are computed for the triple (*x*, *y*, *z*) using the logic (5) proposed by^23^. Note, the computation assumes 8-bit representation for *x*, *y*, and *z*.

5 $$\begin{aligned} \begin{aligned}{}&R_s=\frac{x}{255} \\&G_s=\frac{y}{255} \\&B_s=\frac{z}{255} \\&\mathbf{IF} ~ (R_s \le 0.03928)~ R = \frac{R_s}{12.92} ~ \mathbf{ELSE} ~ R = \left(\frac{R_s+0.055}{1.055}\right)^{2.4} \\&\mathbf{IF} ~(G_s \le 0.03928) ~G = \frac{G_s}{12.92}~ \mathbf{ELSE} ~ G = \left(\frac{G_s+0.055}{1.055}\right)^{2.4} \\&\mathbf{IF} ~ (B_s \le 0.03928)~ B = \frac{B_s}{12.92} ~ \mathbf{ELSE} ~B = \left(\frac{B_s+0.055}{1.055}\right)^{2.4} \end{aligned} \end{aligned}$$

Given the relative luminance *L*, the Color theory provides a way to compute the contrast ratio *CR* between two color triples $E_1$ ($r_1, g_1, b_1$) and $E_1$($r_2, g_2, b_2$). Specifically, we compute first the relative luminance $L_{E1}$ and $L_{E2}$ for respectively $E_1$ and $E_2$ using Eq. (4) and then combine them ($L_{E1}$ and $L_{E2}$) using Eq. (6) adopted from^22^.

6 $$\begin{aligned} CR=\left\{ \begin{array}{ll} \frac{L_{E1}+0.5}{L_{E2}+0.5}, &{} \hbox {If}L_{E1}>L_{E2}; \\ \frac{L_{E2}+0.5}{L_{E1}+0.5}, &{} \hbox {Otherwise;} \\ \end{array} \right. \end{aligned}$$

As mentioned in^22^, the contrast ratio *CR* can assume any value in the range [1...21], where 1 indicates no contrast and 21 indicates the maximum contrast. The controller uses the contrast ratio *CR* and its value range to generate four control signals according to the scheme (7).

7 $$\begin{aligned} Signal= \left\{ \begin{array}{ll} ``Map'', &{} \hbox {If} CR \le 1; \\ ``Map-Distort'', &{} \hbox {If} CR \le 6; \\ ``Distort-Map'', &{} \hbox {If} CR \le 12; \\ ``Skip'', &{} \hbox {Otherwise;} \\ \end{array} \right. \end{aligned}$$

**Table 1:** The control signals generated by the controller.

Signal	Semantic
Map	This signal enables mapping the triple (*u*, *v*, *w*) to the layer without distortion.
Map-Distort	Post-Distortion: this signal enables mapping the triple (*u*, *v*, *w*) to the layer and then distorting the outcome of the mapping.
Distort-Map	Pre-distortion: like Map-Distort except that the triple is distorted first then mapped to the layer.
Skip	The triple skips all subsequent layers and is passed to the output (i.e. no mapping to the current layer nor to the subsequent layers).

**Figure 13:**
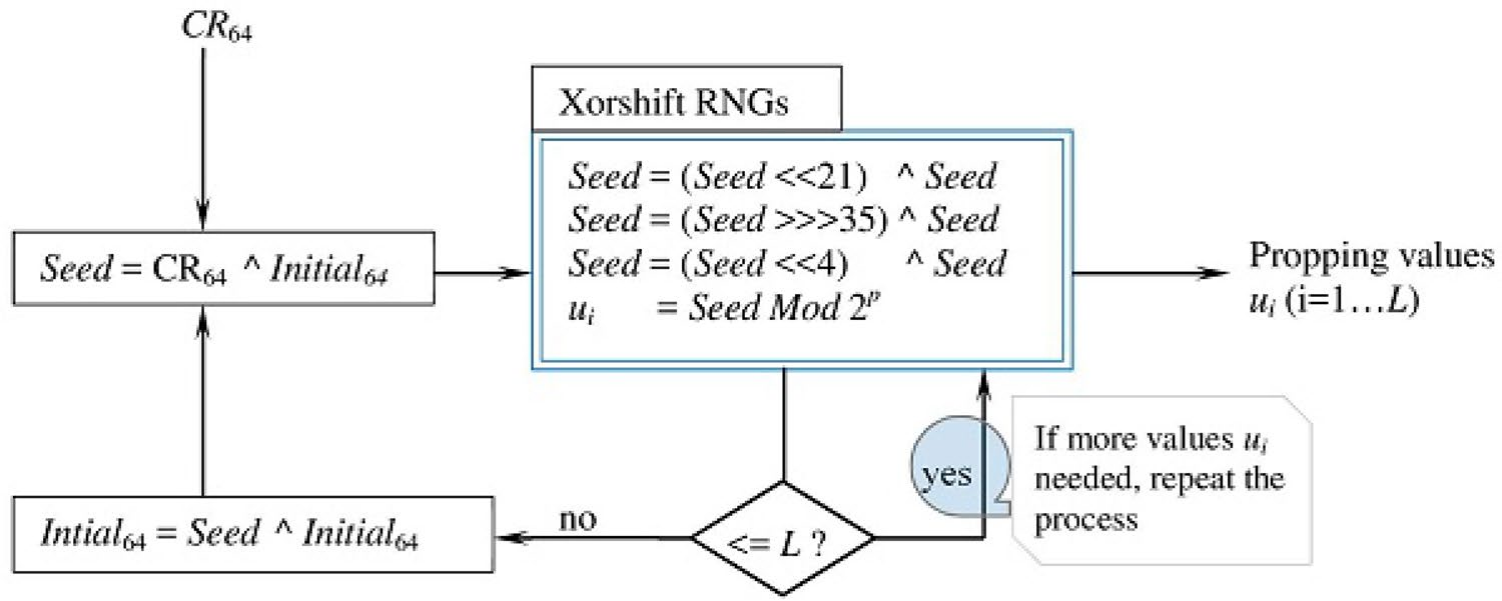
Process for producing the values $u_i$ ($i=1...L$).

In addition to generating the control signals, the controller uses the contrast ratio to generate the values $u_i$ using the logic in Fig. 13. These values are used to support the functionality of the mapping distorter and the diffusion stage. As Fig. 13 shows, the generation of $u_i$ uses the *Xorshift* *RNGs* algorithm^15^, which operates on the input *Seed*. The *Seed* is created by XORing the variables $CR_{64}$ and $Initial_{64}$, where $CR_{64}$ is the leftmost 64 bits extracted from the fraction part of the contrast ratio (*CR*) and $Initial_{64}$ is a 64-bit variable whose initial value is zero. The *Xorshift* *RNGs* is repeated *L* times to produce *L* values $u_i$. After generating the values $u_i (i=1...L)$, the variable $Initial_{64}$ is updated by XORing its current value with the recent value of the *Seed*. This means that generating new values $u_i (i=1...L)$ is necessarily affected by not only the current $CR_{64}$, but also by the previous values of $CR_{64}$, which are accumulated in the variable $Initial_{64}$.

### The mapping distorter

**Figure 14:**
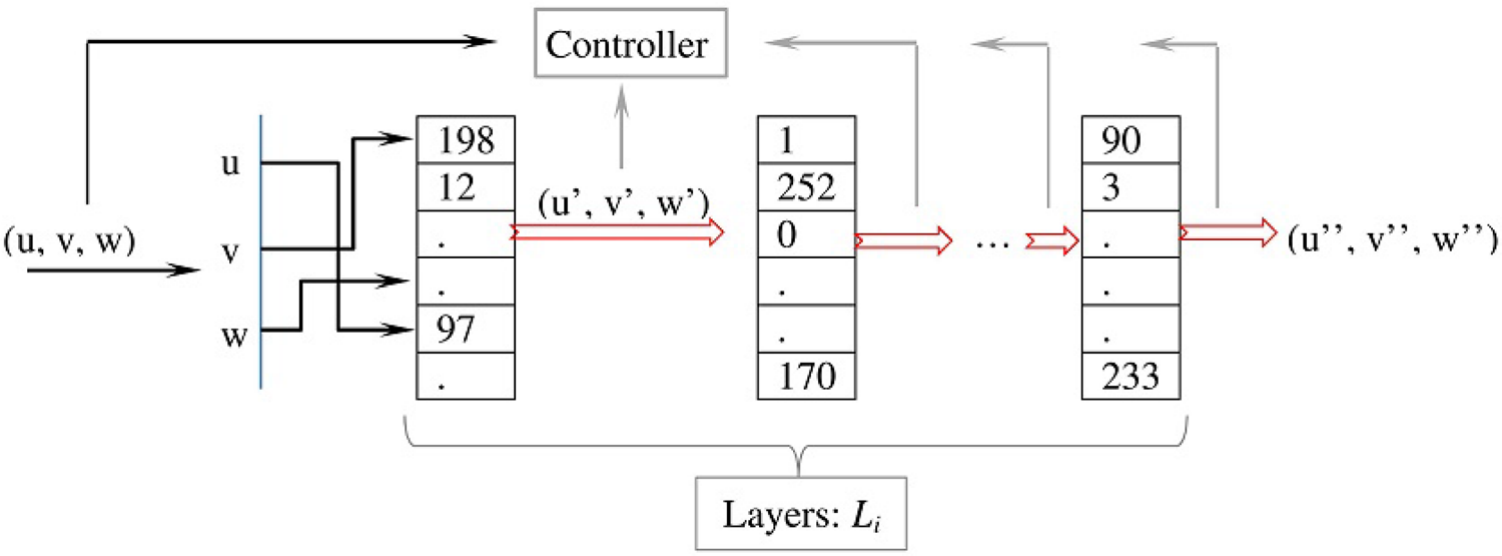
The mapping distorter layers.

The mapping distorter consists of a sequence of *m* layers (see Fig. 14). The output of the current layer is the input for the next one. The connectors between the layers are called mapping links. Each layer $L_i$ consists of $2^p$ cells filled with the integers 0...$2^p$–1, where *p* is the maximum number of bits that represent the used symbols. For instance, if the maximum number of bits is 8, the 2$^8$ cells are populated with the integers 0...255. The entries of each layer $L_i$ are independently scattered using a sequence of chaotic numbers from the chaotic system.

Mapping a triple (*u*, *v*, *w*) to a layer $L_i$ is performed in a natural way: each element of the triple (*u*, *v*, *w*) indexes one of the cells of $L_i$. The content of the indexed cells ($L_i[u]$, $L_i[v]$, $L_i[w]$) yields a new triple ($u', v', w'$), which provides input to both the next layer $L_{i+1}$ and the controller.

The mapping distorter declares a set of distortion operations (Table 2). These operations distort the outcome of the mapping process, making this process non-linear. The distortion operations are placed in a list and reordered using a sequence of chaotic values obtained from the chaotic system. The mapping distorter chooses the operations for manipulating the input triples using three state variables *f*, *s*, and *t*, where these variables select distortion operations to respectively process the first, the second, the third element of the input triple. The initial values for (*f*, *s*, *t*) are zeros. These variables, however, are updated using the scheme (8) before using them for operation selection. (The values$u_1$, $u_2$, $u_3$ are generated using the contract ratio.)

8 $$\begin{aligned} \begin{array}{ll} {} &{} {f} = (({f}<<1) ^\wedge u_1) Mod 2^p \\ {} &{} {s} = (({s}<<1) ^\wedge u_2) Mod 2^p\\ {}&{} {t} = (({t}<<1) ^\wedge u_3) Mod 2^p \\ \end{array} \end{aligned}$$

**Table 2:** The bitwise distortion operations.

Operation	Semantics
Left-Rotate (*x*, *k*)	Left rotate the bits of *x* for *k* positions, where k =1, 2...*p*–1
Mutate ($x, u_5$)	XOR the input symbol *x* with $u_5$
Swap(*x*)	Swap the left half of bits of the input *x* with the right half bits of *x*
FlipSwap($x, u_8, e$)	Flip the left/right half bits of *x* by XORing them with the left/right half bits of $u_8$ and swap the left half bits of *x* with the right half bits. Selecting which half to flip is based on *e* ={0 (left), 1 (right)}
ReplaceWith ($x, u_6$)	Replace *x* with a value $u_6$.
L-ShiftXOR($x, u_7$)	Left shift *x* by 1 position and XOR the result with $u_7$. The resulting integer is confined by taking Module $2^p$

The mapping distorter defines three distortion levels for the input triple (*u*, *v*, *w*). Levels 1, 2, and 3 distort respectively only one element, only two elements, or all the three elements of the triple. Because the triple has three elements, there are 7 different configurations: distort only one element (3 configurations), distort two elements (3 configurations), and distort the three elements (1 configuration). To select any of these configurations, the mapping distorter uses the logic (9). The state variable *q* is initially zero, but updated—prior to any use—using *q* = ((*q*$<<$1) $^\wedge$ $u_4$) Mod $2^p$, where $u_4$ is obtained from the contrast ratio ("The controller" section).

9 $$\begin{aligned} \begin{array}{l} \texttt {Switch} (\texttt {q})\\ ~~\texttt {Case}<(2^q/7)*1: distort~ the~ first~ element ~in~ the~ triple\\ ~~\texttt {Case}<(2^q/7)*2: distort ~the~ second~ element~ in~ the ~triple\\ ~~\texttt {Case}<(2^q/7)*3: distort~ the ~third ~element~ in the triple\\ ~~\texttt {Case}<(2^q/7)*4: distort ~first/second~ elements~ in~ the~ triple\\ ~~\texttt {Case}<(2^q/7)*5: distort~ first/third~ elements~ in ~the~ triple\\ ~~\texttt {Case}<(2^q/7)*6: distort ~second/third~ elements~ in ~the~ triple\\ ~~\texttt {Case} <(2^q/7)*7: distort~ all ~elements~ in ~the~ triple\\ \end{array} \end{aligned}$$

**Figure 15:**
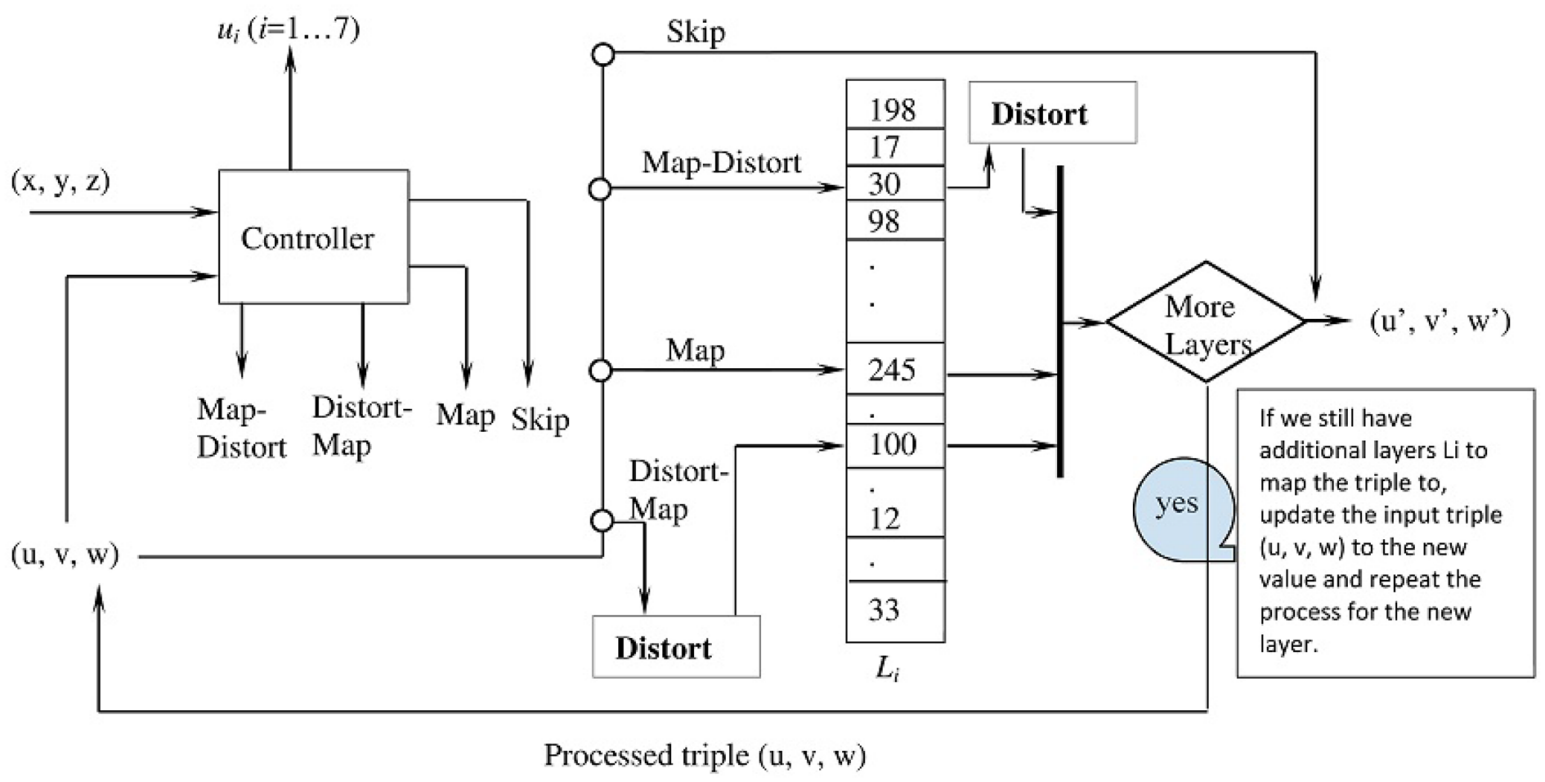
The flow of control of the distortion stage. Note we show only one layer of mapping distorter to simplify the presentation.

After describing each component of the distortion stage, we explain how it works. Figure 15 summarizes the distortion stage flow of control. The input to the distortion stage is two triples (*x*, *y*, *z*) and (*u*, *v*, *w*), where the first triple is directed to the controller while the second is directed to both the controller and mapping distorter. The output of the distortion layer is a new triple ($u', v', w'$).

The controller generates a control signal and produces the values $u_i$ (*i*=1...*L*), where the first four $u_i(i=1...4)$ are used to update the values of the state variables *f*, *s*, *t*, and *q*. The mapping distorter maps the triple (*u*, *v*, *w*) to its layers $L_i$ (*i*=1...*m*) according to the control signal. If the received control signal is Skip, the mapping distorter does not process the input triple (*u*, *v*, *w*) and passes it to the output without modification. If the signal is Map, the triple is mapped to the current layer $L_i$. If the signal is Distort-Map, the mapping distorter uses the state variable *q* to determine the level of the distortion(one, two, or the three elements of the triple) and uses the variables *f*, *s*, and *t* to select the distortion operations. The number of selected operations depends on the level of the distortion. For instance, if the level is to distort three elements of the triple, the variables *f*, *s*, and *t* are used to select three distortion operations. The distorted triple is then mapped to the layer $L_i$ (after being distorted). The same logic applies if the control signal is Map-Distort except that the triple is mapped to the layer $L_i$ and then the outcome of the mapping is distorted.

This process is repeated for each layer $L_i$ of the mapping distorter except if the signal is Skip. (Because the control signal Skip interrupts the mapping and the triple is passed to the output without additional mappings.) Therefore, if there are more layers to map the triple to, the distortion layer updates (*u*, *v*, *w*) to the latest produced value and repeats the processing for the next layer $L_{i+1}$.

## Encryption/decryption process

**Figure 16:**
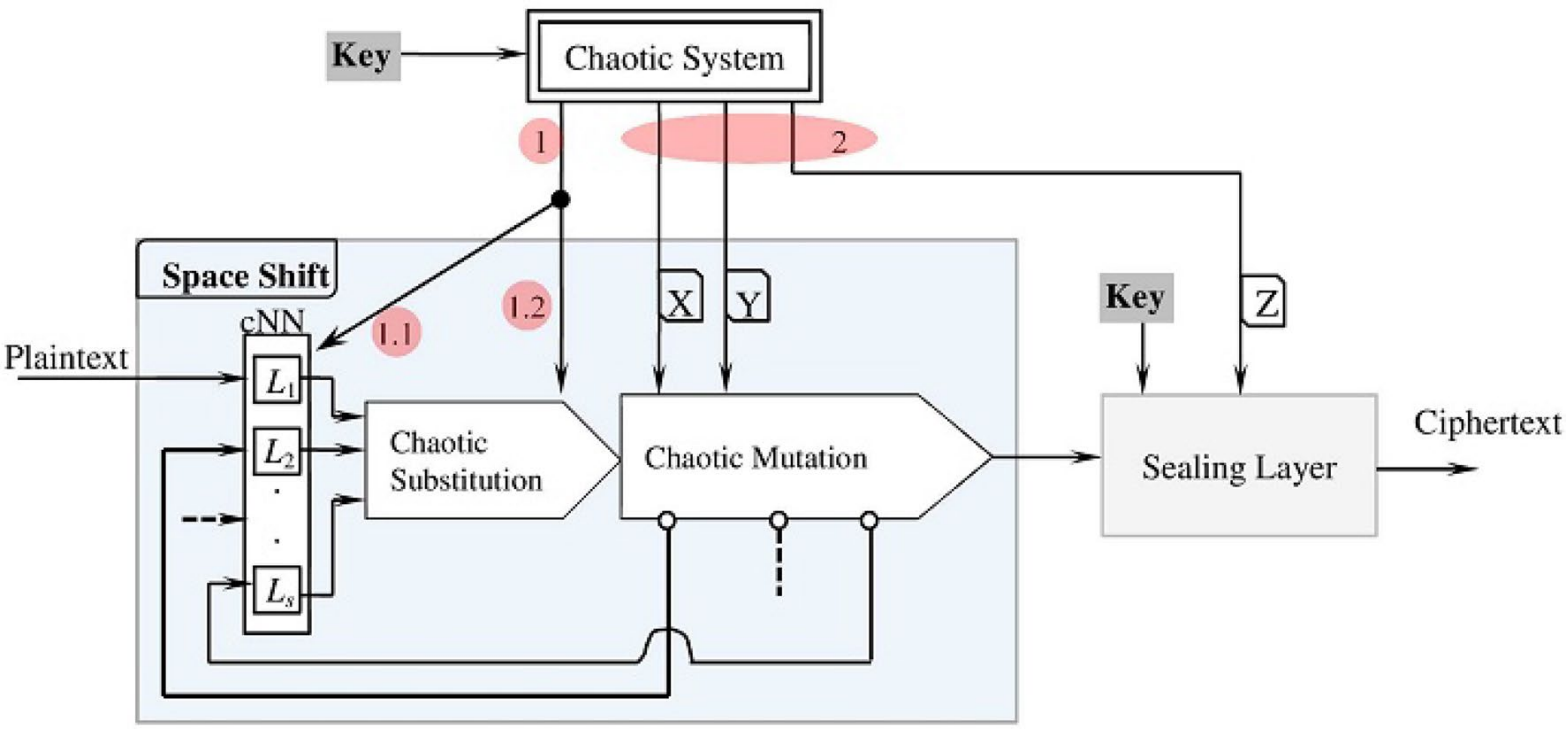
The encryption process.

Figure 16 delineates the proposed encryption technique. The Chaotic System uses the key to generate three streams (*X*, *Y*, *Z*) of chaotic values for supporting the functionality of the encryption different processes. The three streams (*X*, *Y*, *Z*) are combined as to described in “Section Chaotic neural network subprocess (*S*-Layer *cNN*)” for populating the entries of the *cNN* layers $L_i$’s and then for initializing the two parameters (*x*, *m*) of the chaotic substitution map (3). Second, once the *cNN* layers and the parameters (*x*, *m*) are initialized, the streams *X* and *Y* of the chaotic generator are directed to support the operations of Chaotic Mutation subprocess and while the stream *Z* supports the operations of Sealing Layer.

The encryption technique processes the input plaintext in *n*-symbol blocks. The Space Shift processes each block in *s* iterations (*s* is the number of the neural network layers $L_i$). At any iteration *i*, the Space Shift processes the input block using the layer $L_i$ (of the chaotic Neural Network, *cNN*) and then distorts the output of the layer $L_i$ using Chaotic Substitution and Chaotic Mutation operations. Because both the chaotic substitution and chaotic mutation are nonlinear and chaotic, they have a very sophisticated computational behavior that highly confuses the input. The output of the chaotic shift receives further confusion by the sealing layer. The sealing layer hides the output symbols by XORing them with enormously complicated key-dependent codes.

**Figure 17:**
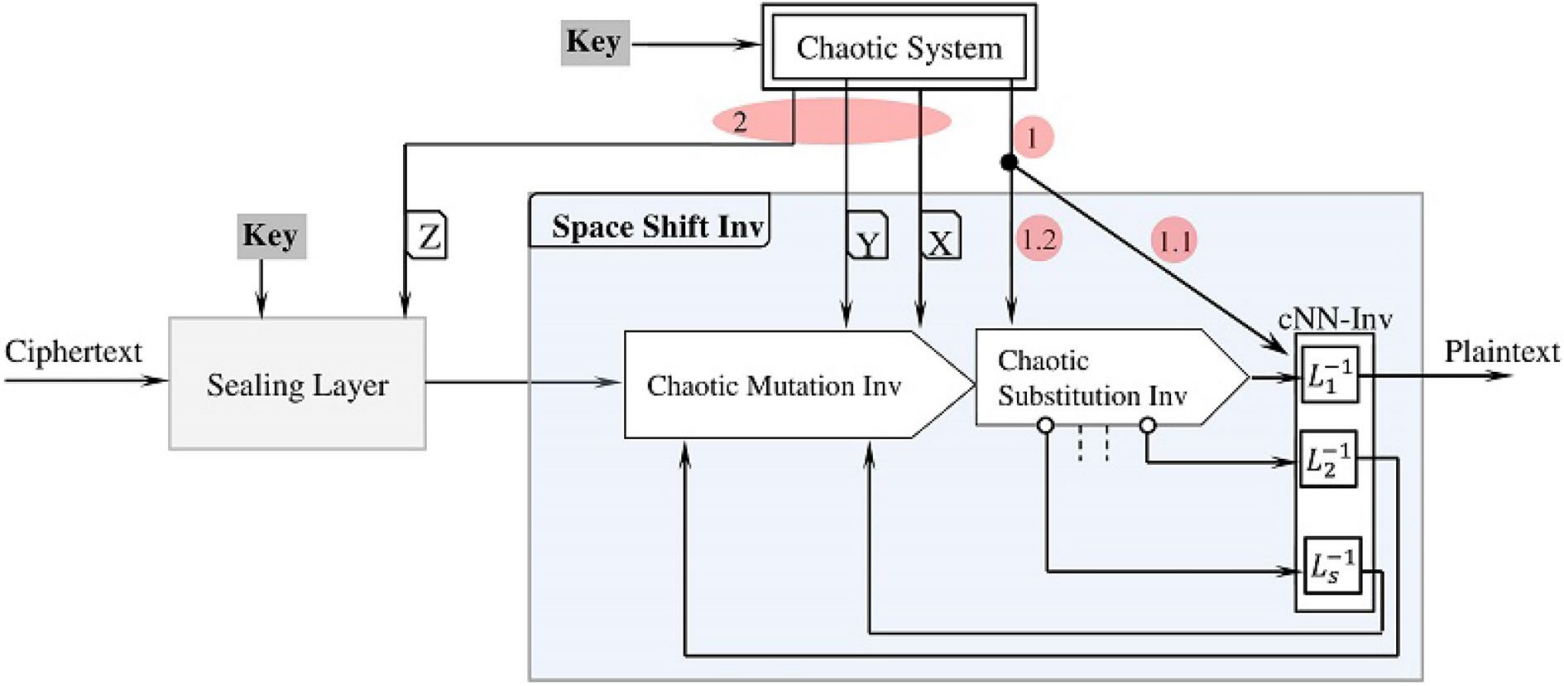
The decryption process.

Figure 17 shows the decryption process. The initialization step follows the same steps of the encryption except for the need to compute the inverse of the layers of the chaotic neural network. Once the process is initialized, the decryption process restores the plaintext block using the inverse of the encryption operations (see Fig. 17). The decryption process first handles the ciphertext block using the Sealing Layer to remove the key impact. The output of the Sealing Layer goes through a sequence of operations: Chaotic Mutation Inv$\rightharpoonup$Chaotic Substitution Inv$\rightharpoonup$cNN-Inv layer. Each Inverse operation removes the impact of the corresponding encryption operation on the block. Note, the decryption process applies the layers of the “cNN-Inv” backwards: from the layer $L_s^{-1}$ down to the layer $L_1^{-1}$.

## Performance analysis

We evaluate in this section the performance of the proposed technique. We first analyze the important properties of the sealing code layer. Specifically, we analyze the entropy, avalanche effect, and the randomness of the sealing codes. These are very important properties and the sealing code must have high entropy, high avalanche effect, and random to be effective. We next evaluate the performance of the encryption technique by running different security tests on the ciphertext.

### Sealing code performance

The test case consists of a large set of 128-bit keys. For better matching the possible distribution of the keys in real-world applications, we used 2500 random keys generated using the service (passwordsgenerator.net), 170 handcrafted keys, 3278 low entropy keys. The low entropy keys (3278) were generated using an input key of all zeros as follows. We created 128 keys by flipping only the $i^{th}$ bit of the input key (*i*=1...128). The rest of the keys are created by flipping *j* bits at random positions of the input key (*j*=2, 3, 4, 5...64). By flipping only up to half of the key, we kept the resulting keys with low entropy.

**Figure 18:**
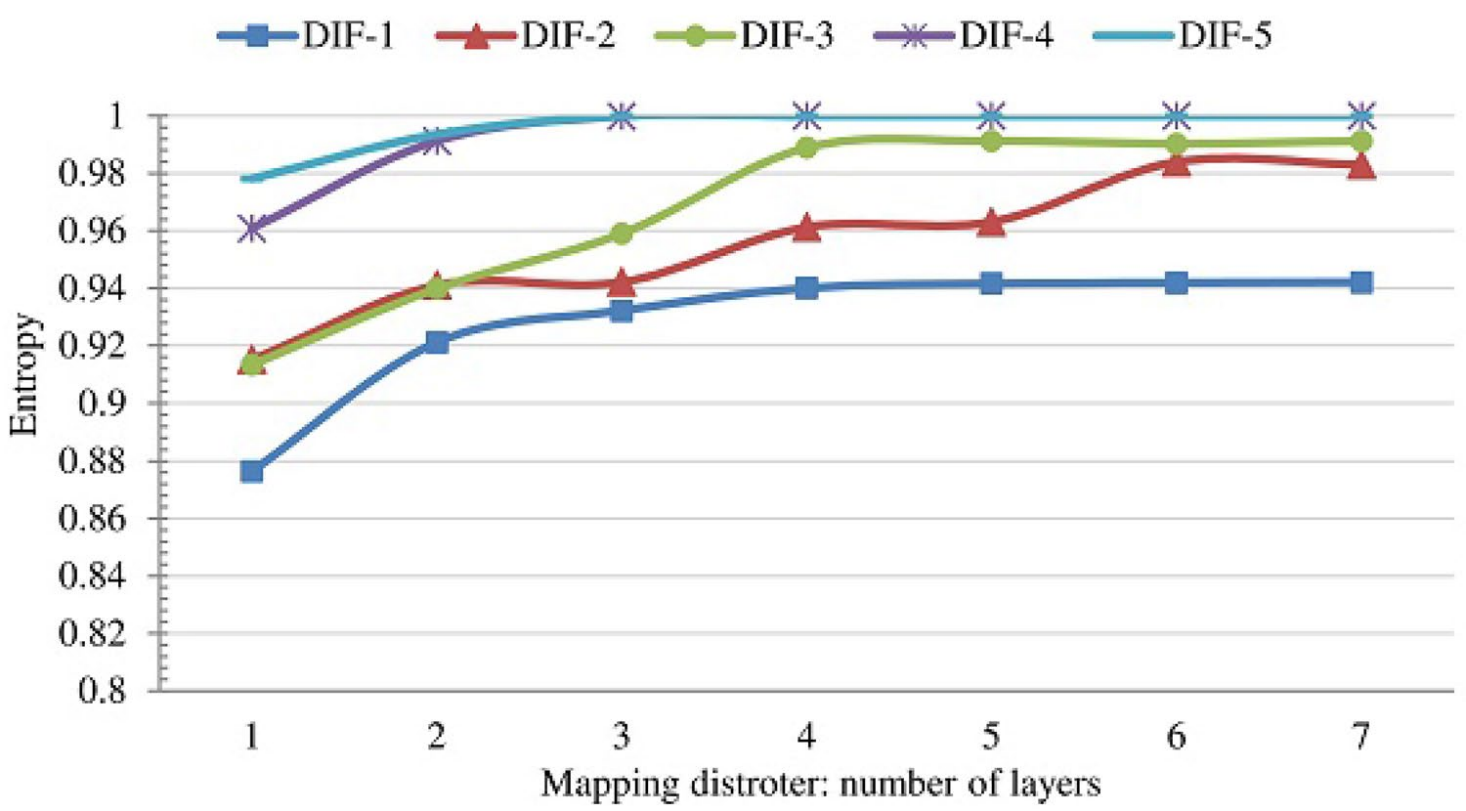
The entropy as a function of number of diffuse stage rounds (DIF-*i*) and the number of mapping distorter layers.

**Figure 19:**
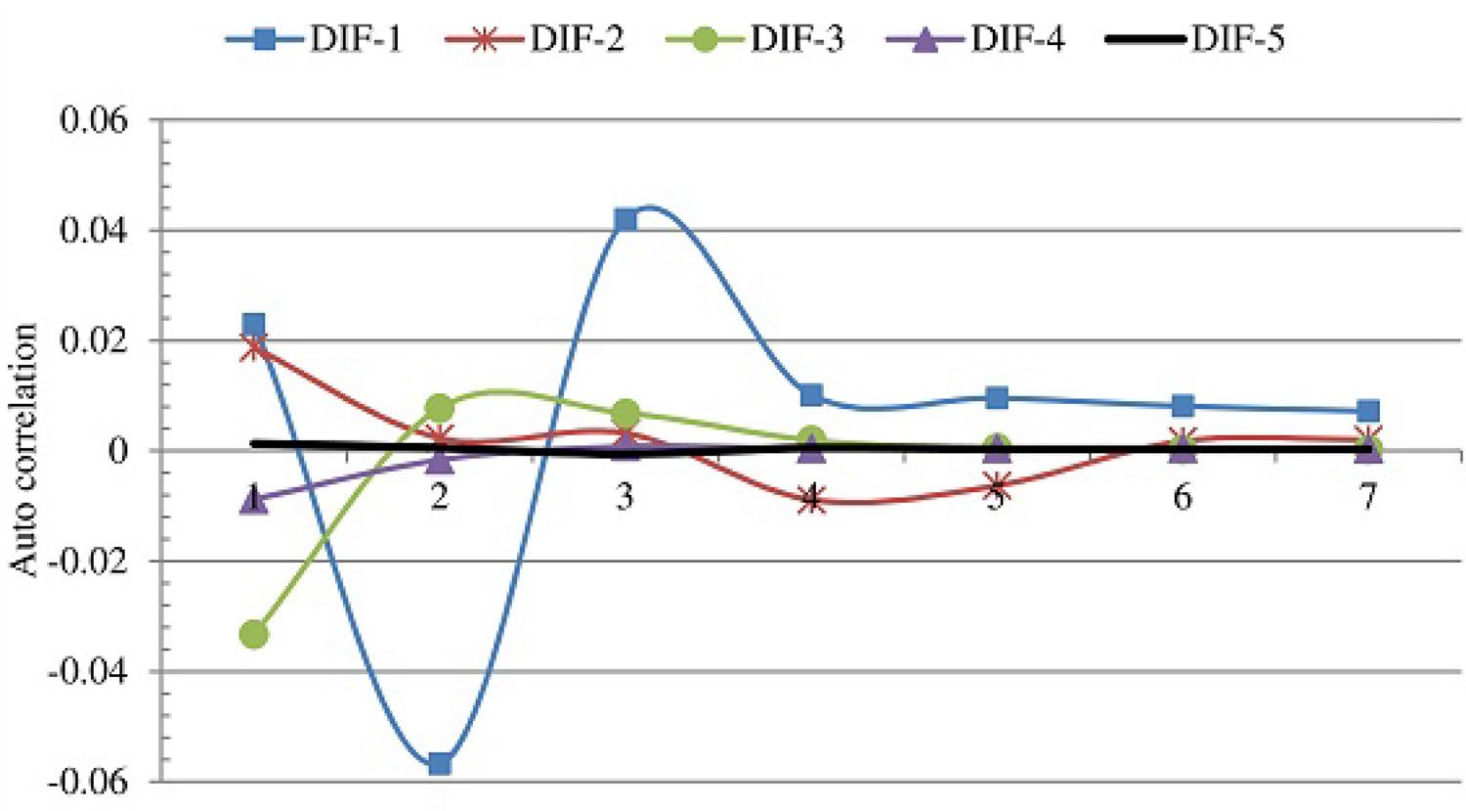
The autocorrelation as a function of number of round (DIF-*i*) and the number of mapping distorter layers.

The sealing code layer used each of these keys to generate large sealing code sequences. Each sequence is of 128,000 symbols—each symbol is 8 bits. Since the execution of the diffuse stage and the mapping distorter (two subprocesses in the sealing layer) depends on the number of rounds and the number of mapping layers, we analyzed the impact of rounds and the mapping layers on the overall performance of the sealing layer. Therefore, the sealing layer was executed several times for different values of the rounds and the mapping distorter layers. Figures 18 and 19 shows the performance of the sealing layer in terms of two important performance metrics: entropy (Fig. 18) and autocorrelation (Figs. 19). The numbers represent the average over all the sequences. The figures show a general improvement pattern: the values of the entropy improve (getting closer to the ideal value 1) and the values of the autocorrelation improve (getting closer to 0) as the number of rounds and the number of mapping distorter layers increase. However, examining the figures, one can see that there is no significant improvement beyond the 4 round (DIF-4) and 4 mapping distorter layers. Since increasing the number of rounds and mapping layers entails an increase in the execution time, we fixed the number of rounds to 4 and the number of mapping layers to 4. We refer to this configuration by the effective configuration.

Table 3 shows the results of the ENT random test on the sequences that were generated using 4 rounds for the diffuse stage and 4 layers of the mapping distorter. The table shows the result of five important ENT randomness tests. The entropy is close to 1 (the ideal values for bit sequence), the Chi-square values indicate that the sequences are random, the estimation for $\pi$ is close to the actual value with a tiny error (please see^24^ for ENT test values interpretation). The serial correlation coefficient is sufficiently small (close to 0) and the arithmetic mean is close to the ideal value 0.5. These ENT test results indicate that the sequences generated by the sealing layer does not deviate from random.

**Table 3:** ENT’s randomness tests.

Randomness Test	Test output (stream of bits)
Entropy	0.9999982
Chi-square Test	54.531%
Arithmetic Mean	0.50785
Monte Carlo Value for Pi ($\pi$)	3.1414260 (Err. −6.7$\times 10^{-5}$)
Serial Correlation Coefficient	−0.00056

To effectively examine the avalanche effect of the proposed technique, we used a low entropy 128-bit key of all zeros. We then constructed different perturbed keys by flipping bits at random positions of the low entropy input key. Due to the prohibitively large possibilities, the number of flipped bits is 1, 2, 3, 4, 8, 12, 16, 24, 32, 64, 96. For each number of flipped bits, we constructed 20 perturbed keys. For instance, we constructed different 20 perturbed keys, where each key is created by flipping the input key in a single random position. The sealing code layer produced, for each key, a code sequence of 1024 symbols (8192 bits). In addition, when the sealing code layer produced the code sequence, it used the effective configuration (4 rounds for the diffuse stage and 4 layer for the mapping distorter). We computed Hamming distance between the sequence generated using the input key and the sequences generated using its corresponding perturbed keys. (Hamming distance is the number of bit differences between two sequences at the corresponding positions).

**Figure 20:**
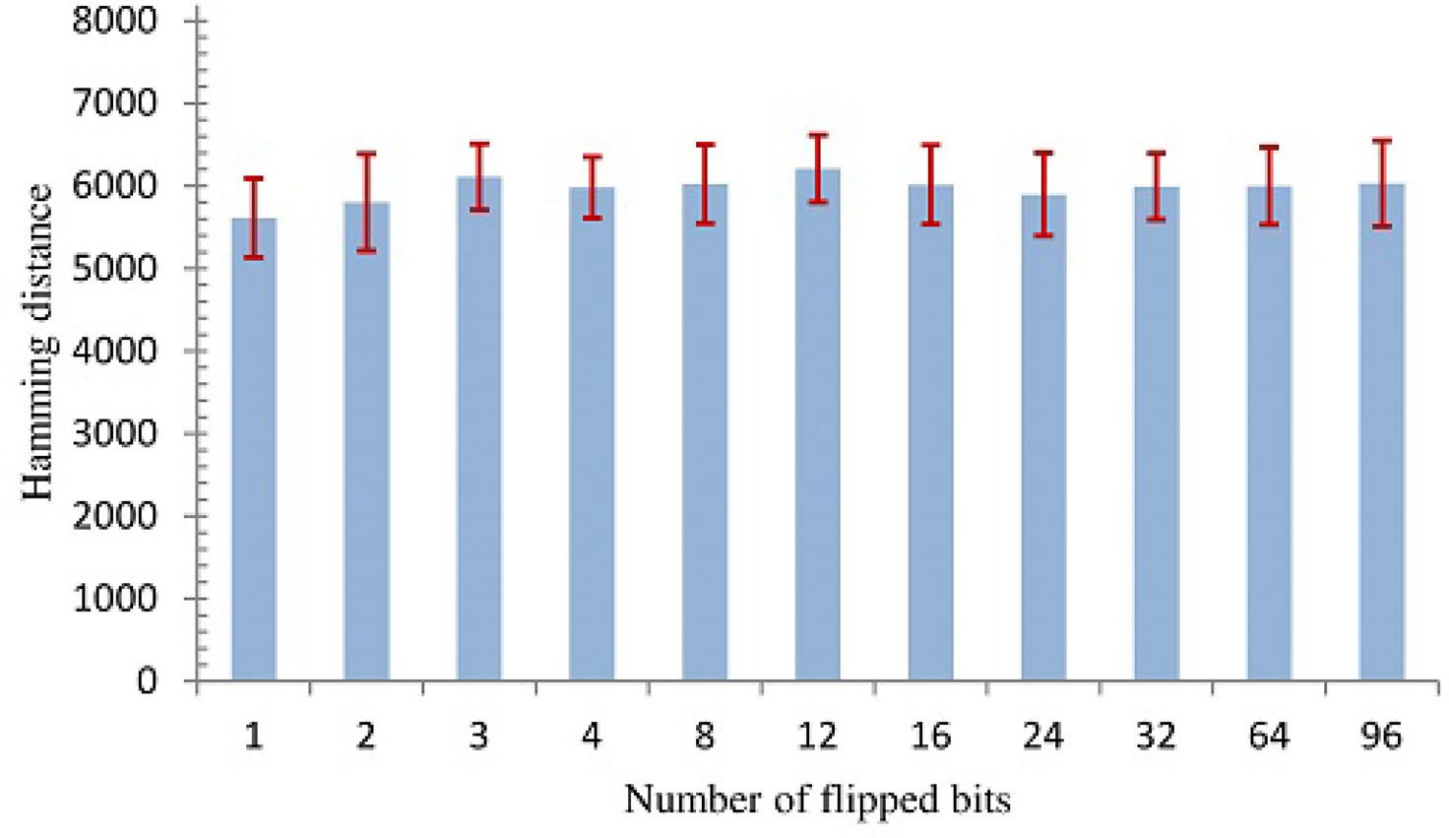
The average avalanche effect as a function of number of flipped bits.

Figure 20 shows the average avalanche effect based on the number of flipped bits. As the figure shows, the minimum average avalanche exceeds 5000 bits difference. This means when we change a bit or more in the input key, the bit difference between the sequence generated from the input key and the sequence generated from its perturbed key exceeds half of the length of the sequence (8,192 bits). The confidence intervals around the average—represented by the error bars—show that there is no significant difference in the average of bit difference when the number of the flipped bits changes. These numbers are indicative and show that the sealing code layer has a large avalanche effect.

### Security analysis

This section presents the performance analysis of the proposed encryption technique. Successful testing considers all the parameters that impact the performance of the technique. These parameters include key variations, plaintext variations, and the entropy of the plaintext and the key. National Institute for Standards and Technology has provided a well-designed framework for thoroughly examining the performance of encryption techniques^25,26^, which include the following three sets of data.

1. *Key Avalanche Data Set* Examines the impact of the key’s changes on the randomness of the resulting ciphertext (fixed plaintext).
2. *Plaintext Avalanche Data Set* Examines the impact of the plaintext’s changes on the resulting ciphertext (fixed key).
3. *Plaintext/Ciphertext Correlation Data Set* Examines the correlation between plaintext/ciphertext pairs (high correlation means bad security).

We created the above three sets consistently with the specification^25^. For the key avalanche, we created and analyzed 1200 sequences of size 524,288 bits each. We used a fixed low entropy 4096-bit plaintext of all zeros and 1200 random keys each of size 128 bits. Each sequence was constructed by concatenating 128 derived blocks created as follows. Each derived block is constructed by XORing the ciphertext created using the fixed plaintext and the 128-bit key with the ciphertext created using the fixed plaintext and the perturbed random 128-bit key with the *i*th bit modified, for $1\le i\le 128$. For plaintext avalanche, we created and analyzed 1200 sequences of size 524,288 bits each. The construction of the sequences is similar to the construction of the key avalanche set except we used 1200 random plaintexts of size 4096 bits and a fixed low entropy 128-bit key of all zeros. For plaintext/ciphertext correlation, we constructed 800 sequences of size 614,400 bits. Each sequence is created as follows. Given a random 128-bit key and 1200 random plaintext 512-bit blocks, a binary sequence was constructed by concatenating 1200 derived blocks. A derived block is created by XORing the plaintext block and its respective ciphertext block. Using the 1200 (previously selected) plaintext blocks, the process is repeated 799 times (one time for every additional 128-bit key).

We ran the NIST battery of randomness test on the above three data sets. Tables 4 and 5 show the results presented in terms of the number of sequences that passed a specific test (Success) and their percentages (Rate%). The used level of significance is $\alpha =0.05$, which means ideally no more than 5 sequences out of 100 may fail a corresponding test. In practice, however, any set of data is likely to deviate from this ideal. To consider this, we computed an upper bound on the number of the sequences that may fail using the formula (10)^25^. (In formula (10), *S* is the total number of sequences and $\alpha$ is the level of significance.) The upper bounds are presented in the column “M.Fail”.

The performance numbers in Tables 4 and  5 showed a high percentage pass. More than 96% of the key/plaintext data set sequences and more than 95% of the plaintext/ciphertext data set sequences passed the NIST randomness tests. The number of sequences that failed any of the randomness tests is much less than the maximum number that is expected at a significance level of 0.05.

10 $$\begin{aligned} Max~Failure = S.(\alpha +3.\sqrt{\frac{\alpha (1-\alpha )}{S}}) \end{aligned}$$

**Table 4:** NIST’s random test figures: Key/Plaintext Avalanche.

Test	Key Avalanche	Plaintext Avalanche	M.Fail
	Success (Rate%)	Success (Rate%)	
Runs	1200 (100%)	1200 (100%)	82.65
Monobit	1192 (99.3%)	1200 (100%)	82.65
Spectral	1156 (96.3%)	1162 (96.8%)	82.65
Serial	1189 (99.08%)	1200 (100%)	82.65
Cumulative Sums	1172 (97.6%)	1165 (97.1%)	82.65
Non-Overlapping Template Matching	1196 (99.7%)	1196 (99.7%)	82.65
Overlapping Template Matching	1186 (98.8%)	1188 (99%)	82.65
Linear Complexity	1200 (100%)	1183 (98.5%)	82.65
Binary Matrix Rank	1184 (98.7%)	1178 (98.2%)	82.65
Maurer’s “Universal Statistical”	1181 (98.4%)	1185 (98.6%)	82.65
Approximate Entropy	1193 (99.4%)	1193 (99.4%)	82.65
Longest Runs of Ones in a Black	1191 (99.2%)	1195 (99.6%)	82.65

**Table 5:** NIST’s random test figures: Plaintext/Cipheredtext Correlation.

Test	Success (Rate%)	Max Failure
Runs	798 (99.7%)	55.1
Monobit	800 (100%)	55.1
Spectral	765 (95.6%)	55.1
Serial	788 (98.5%)	55.1
Cumulative Sums	781 (97.6%)	55.1
Non-Overlapping Template Matching	775 (96.9%)	55.1
Overlapping Template Matching	792 (99.0%)	55.1
Linear Complexity	776 (97.0%)	55.1
Binary Matrix Rank	776 (97.0%)	55.1
Maurer’s “Universal Statistical”	783 (97.9%)	55.1
Approximate Entropy	789 (98.6%)	55.1
Longest Runs of Ones in a Black	798 (99.7%)	55.1

Tables 6, 7, and 8 show the ENT battery of randomness tests results. These tests give additional insights about the performance of the proposed technique. Referring to these tables, the entropy is so close to 1 (ideal value is 1). The arithmetic mean is close to 0.5 (ideal exactly 0.5). Having a very close approximation for the $\pi$, with an error magnitude of $10^{-4}$, using Monte Carlo method indicates very good randomness of the sequences. Finally, the serial correlation is so close to zero (ideally zero), which statistically indicates that there is no dependency between a bit and its predecessor.

**Table 6:** ENT Test results (Key Avalanche).

ENT Test	Average value	Min/Max value
Entropy	0.999998	0.9997901/1.00
Chi-square	57.098	53.133/62.453
Arithmetic Mean	0.5003538	0.4918323/0.5035560
Monte Carlo estimation of $\pi$	Err. 2.9E−4	Err. 1.87E−6/3.33E−3
Serial Correlation	4.1E−5	3.9E−6/2.44E−4

**Table 7:** ENT Test results (Plaintext Avalanche).

ENT Test	Average value	Min/Max value
Entropy	0.999988	0.9998475/1.00
Chi-square	61.199	57.995/63.333
Arithmetic Mean	0.4998363	0.4989756/0.5099671
Monte Carlo estimation of $\pi$	Err. 6.08E−4	Err. 1.0E−5/5.23E−3
Serial Correlation	8.7E−5	1.7E−6/8.76E−4

**Table 8:** ENT Test results (Plaintext/Ciphertext Correlation).

ENT Test	Average value	Min/Max value
Entropy	0.9999899	0.9997601/1.00
Chi-square	60.053	59.111/64.377
Monte Carlo estimation of $\pi$	Err. 4.7E−4	Err. 6.3E−4/8.072E−4
Serial Correlation	5.01E−5	1.20E−6/7.104E$-3$

### Security attacks resistance

We analytically demonstrate that the proposed technique is effective against critical types of attacks. We specifically show why the proposed encryption method can resist deferential and classic attacks.

#### Deferential attacks

Differential attacks are so effective and very challenging to encryption techniques. They can exploit vulnerabilities due to the lack of effective confusion that can hide the key identity. Therefore, if the encryption technique does not induce enough confusion to hide the patterns of the key, attackers can flip one or more bits in plaintext and observe the differences in the values of the bits between the two ciphertexts to identify a pattern that helps crack the encryption technique^27^. The proposed encryption technique uses the key either to initialize the chaotic system parameters or to generate the sealing code. The initialization process ("Chaotic system: Lorenz chaotic system" section) applies nonlinear operations to generate initialization values. These operations ensure deep manipulations of the key through key splitting and constant randomization-update operation. The key trace (pattern) is consequently eliminated. The sealing code layer also uses the key. The key is deeply processed using a one-way expansion algorithm and a distortion operation whose functionality is controlled by color theory principles. As analyzed in "Sealing code performance" section, the output of the sealing layer has very high entropy, is random, and has a high avalanche effect (flipping a bit forces more than $\frac{1}{2}$ of the output bits to change).

#### Classic attacks

Classic attacks involve four powerful types: ciphertext-only, known-plaintext, chosen-plaintext, and chosen-ciphertext attacks. Although all of these attacks can seriously threaten the encryption technique, chosen-plaintext attack is the most effective one^27–29^. If the encryption technique can resist this attack, it can resist the others^30^.

The proposed encryption technique resists the chosen-plaintext attacks due to both the space chaotic shift and the sealing layer. The space chaotic shift processes plaintext using three nonlinear subprocesses . The chaotic neural network subprocess makes deep changes using chaotic numbers and irreducible operations of Galois field. The chaotic substitution intensifies the confusion through nonlinear substitution for the symbols of the input. In particular, the chaotic substitution subprocess uses plaintext to induce chaos in the substitution due to the enhanced logistic map equation. The chaotic mutation imposes chaotic and fine-grained changes to the symbols. The output, therefore, is sharply different and not correlated to the input due to the accumulated confusion induced by these three nonlinear operations. The sealing code layer produced key-based code sequences that are random with high entropy and avalanche effect. The contribution of the sealing code layer is additional confusion resulted from the variations of the key. This additional confusion makes it impossible for hacking techniques to locate and follow any patterns that may lead to knowing the input plaintext.

### Time complexity analysis

The major merit of the proposed technique is that the two principal components—the space chaotic shift and the sealing layer—are completely independent. They can work in a parallel mode. Therefore, we analyze the time complexity for each and consider the maximum complexity of the two as the complexity of the technique.

The space chaotic shift process consists of three subprocesses. The chaotic neural network subprocess multiplies a matrix with a vector of *n* entries (the input block). The complexity of this multiplication is *O*(*n*), where *n* is the input size. The chaotic substitution subprocess handles its input block (*n* symbols) using simple operations—*XOR*, integer multiplication, and logical shift. Each of these simple operations is *O*(1) and consequently, the total time is *O*(*n*). The chaotic mutation subprocess includes operations that access the mesh by directly indexing a cell or two, *XOR* operation, and multiplication under Galois field. These operations are simple and each is *O*(1). We have additionally a one-time initialization for the neural network nodes and for initializing the Lorenz chaotic system parameters. These operations are linear and upper bounded by *O*(*n*). Based on this, the complexity of the space chaotic process is linear and has an upper bound of *O*(*n*).

The sealing layer process consists of also three subprocesses. The diffusion stage executes *XOR*, logical shift, Galois field multiplication. These operations are simple and each is *O*(1). Therefore, for an input block of *n* symbols, the complexity of the diffusion stage is upper bounded by *O*(*n*). The input expansion stage uses SHA-512 hashing algorithm. The algorithm is known for its time efficiency and therefore its complexity is upper bounded by *O*(*n*). The distortion stage involves integer value arithmetic operations (addition, division, and multiplication) each of *O*(1). It also involves *XOR*, logical shift, and direct indexing for the mapping distorter layers. These operations, however, are simple and of *O*(1). The one-time initialization for the mapping distorter layers is of *O*(m), where *m* is the size of each layer (the size of each layer is typically small and of 256 integers). Accordingly, the complexity of the sealing layer is linear and upper bounded by *O*(*n*).

**Figure 21:**
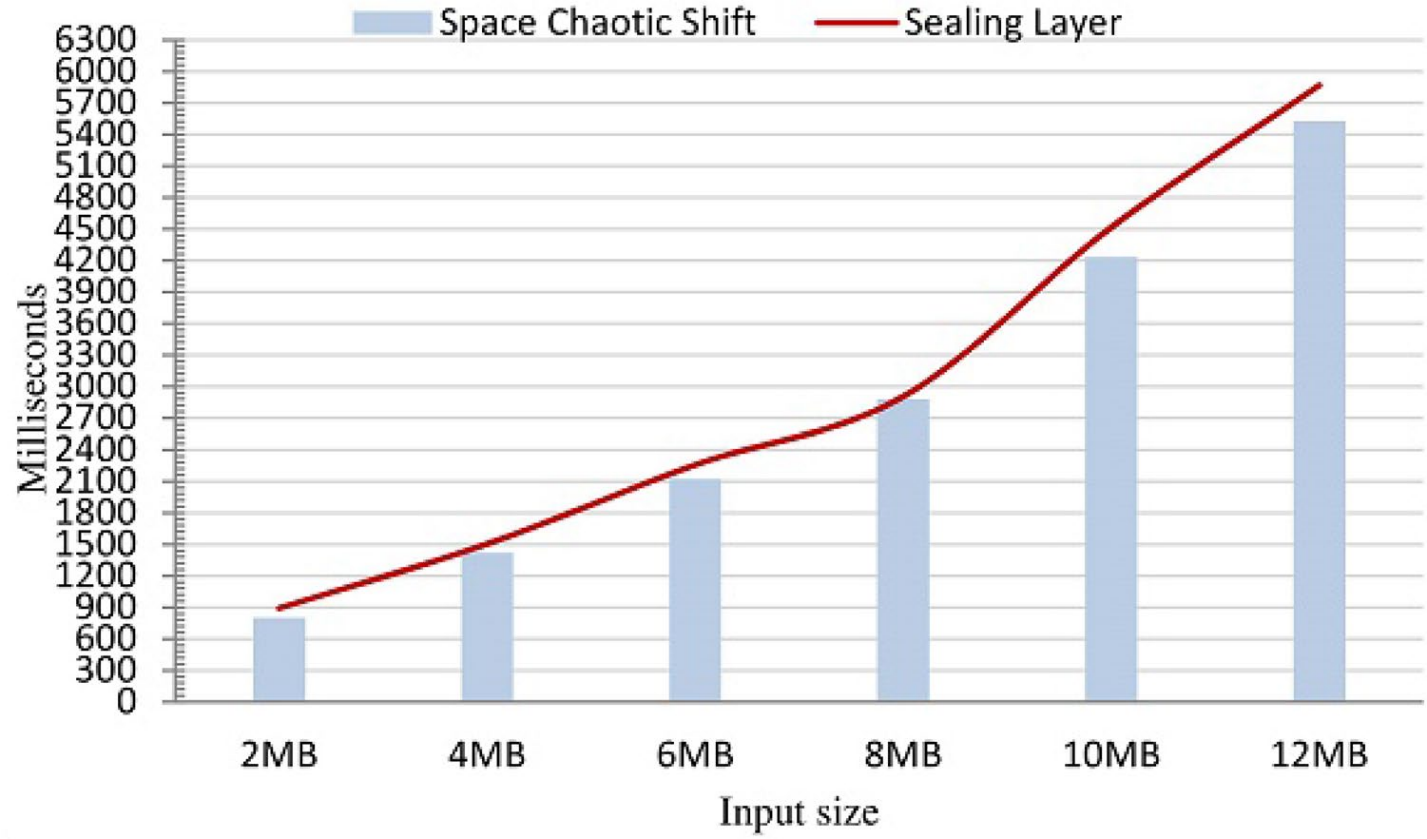
The time (milliseconds) for processing different input sizes.

According to the definition of Big-*O*, the time complexity of the proposed technique is *O*(*n*). To the best of our knowledge, there is no encryption technique whose computational model is less than *O*(*n*) (including AES^19^). Figure 21 shows the time required for different input sizes (megabytes). The hardware specification was an Intel Core *i*5 with 4GB memory and windows 10 (OS). The bars represent the time required by the space chaotic shift process to produce its final input. The curve represents the time required by the sealing layer to generate a sealing code sequence of the same size as the ciphertext. The curve and the bars are increasing roughly linearly. This is consistent to a large extent with the complexity analysis. Although the figure shows that the sealing layer requires a bit more processing time than the space chaotic shift, this extra time is not significant.

## Discussion

We discuss now the experimental results presented in "Performance analysis" section. Based on the results in Tables 4 and 5, the output (ciphertext) of the proposed technique passed the NIST randomness tests due to the high percentage of sequences that passed each randomness test and the low parentage of sequences that failed. Referring to Tables 4 and 5, one can see that the number of sequences that failed an individual test is less than the maximum number of sequences that may fail a test (as estimated by the NIST formula 10). Additionally, the results of ENT randomness tests shown in Tables 6, 7, and 8 indicate that the method output is random. The well-established NIST framework, which was used to test the standard encryption technique competition, states that encryption techniques whose output passes the defined randomness tests are considered effective. Accordingly, since the proposed technique passed all the randomness tests with the number of failed sequences less than the maximum number of sequences that may fail, it then is effective.

The experimental analysis of the effectiveness of the proposed technique is supported also by the theoretical analysis ("Security attacks resistance" section). The high entropy and avalanche effect of the key sequence generated by the sealing layer ensure that the key is secure^27^. Furthermore, we showed in "Security attacks resistance" section that our technique can resist a chosen-plaintext attack. Based on^28,29^, if the technique can resist a chosen-plaintext attack, it can resist all other types of attacks.

According to our discussion in "Time complexity analysis" section, the technique is time-efficient. The theoretical analysis for the time showed that the technique has linear time complexity. This is indicated by the upper limit of the time complexity *O*(*n*). This theoretical analysis (in terms of Big-O) is well supported by the time measurements in Fig. 21, which plots the time requirement for encrypting text files of varying sizes. One can observe that the time required for encrypting each file increases as the size of the file increases, but this time increase is pretty linear.

**Figure 22:**
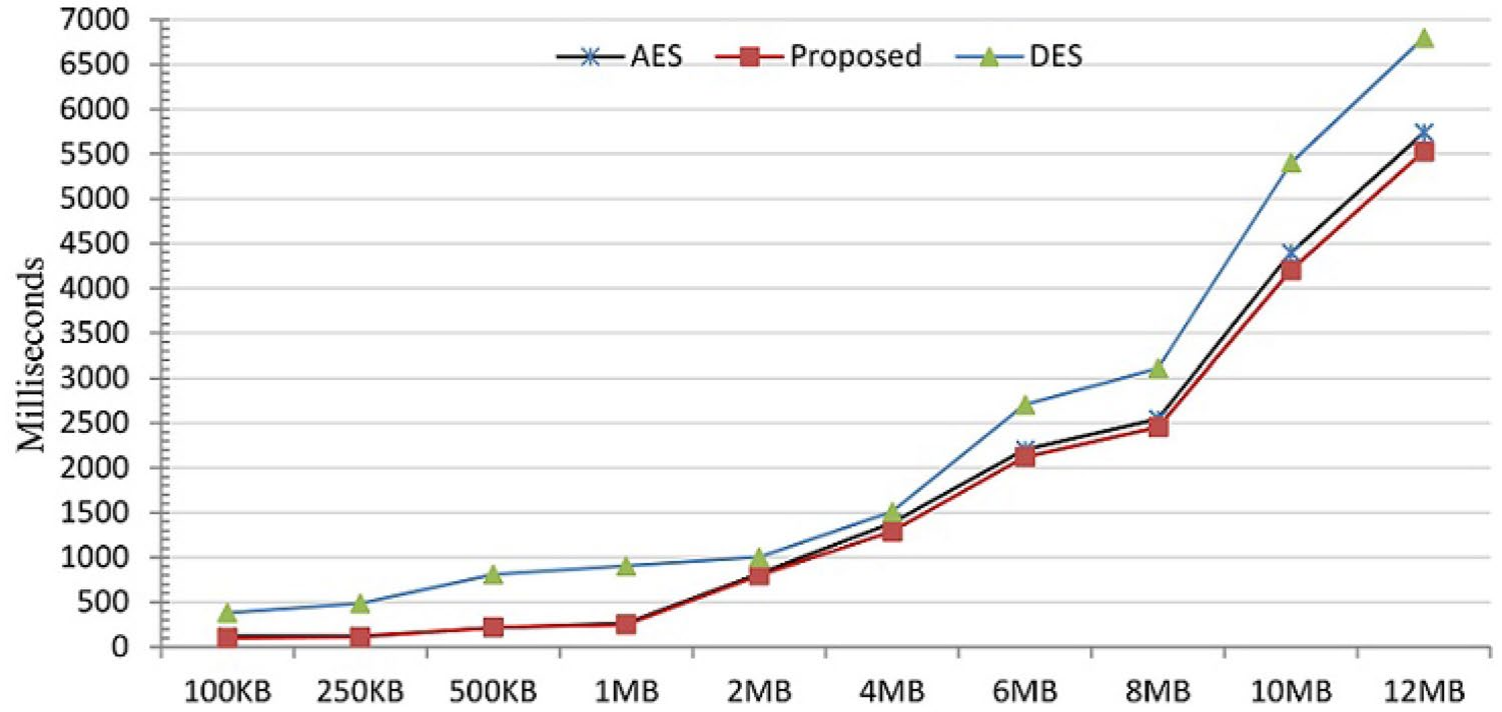
The execution time for proposed, AES, and DES for different input sizes.

**Table 9:** The execution time for proposed, AES, and DES for different input sizes

Input size	AES	Proposed	DES
100KB	120	98	380
250KB	123	111	487
500KB	209	218	811
1MB	262	251	905
2MB	817	800	1001
4MB	1388	1289	1509
6MB	2203	2122	2705
8MB	2544	2455	3111
10MB	4397	4203	5400
12MB	5737	5528	6799

To further examine the time performance of the proposed technique, we compared it to state-of-the-art encryption techniques. In particular, we used two standard algorithms AES and DES as a base for time performance comparison. Because the proposed technique was implemented in Java, we also used the Java implementation of AES and DES so that we have similar software platform. The hardware platform was an Intel Core i5, 4GB memory, and Windows 10. We executed the proposed technique and the two baseline techniques on inputs with different sizes. (The techniques AES and DES were executed using ECB mode.) For each input, we executed each technique 20 times and recorded the average over the 20 runs. Figure 22 plots the average execution time for the proposed technique and the two baseline techniques. (Table 9 shows the average execution time numbers rounded to the closest integer). As can be seen, DES required longer time than AES and the proposed technique for all the input sizes. The proposed technique and AES required almost the same time for input sizes up to 2MB. However, the execution time of the proposed technique becomes slightly better (shorter) than the execution time for AES for larger input sizes (>2MB). These numbers indicate that the proposed technique has an execution time similar to the time required by state-of-the-art techniques and can be widely used for real-world applications. Although it would be more comprehensive to compare the proposed technique with more encryption techniques, we think that the comparison with AES and DES provides sufficient evidence for the time efficiency of the proposed technique. (AES and DES are standard, have an efficient implementation in Java platform, and AES is known for its execution time efficiency^31^.)

## Related works

This section compares the proposed technique to state-of-the-art techniques. In the comparison, we show the properties of the computational model of each technique and then compare these models with the computational model of the proposed technique.

Chaotic systems provide a significant foundation for cryptographic applications due to their hypersensitivity to the initial conditions and input parameters. Slight modifications to the initial conditions or the inputs produce unpredictable behavior^32,33^. Although chaotic systems are mostly used in image cryptosystems, their hypersensitivity and unpredictability are certainly applicable to text cryptosystems^34,35^. The techniques^17,36–40^ used chaotic systems for inducing enough confusion in the resulting ciphered image. The authors^41^ proposed a color image encryption technique based on one-time keys and robust chaotic maps. This technique used a linear piecewise chaotic map to generate the keys with a real random generator. The proposed technique^29^ used cyclic shift and sorting to permutate the image bits and produce high confusion. The proposed technique^42^ used dynamic piecewise coupled mapping lattice, which generates good chaotic behavior that induces large confusion in the resulting ciphered image. In^43^, the proposed image encryption technique used a semi-tensor product matrix along with a Boolean network as fundamental operations for encrypting images. Fractal Sorting matrix (FSM) with global chaotic pixel diffusion is proposed in^44^. Based on the FSM and global chaotic pixel diffusion, this paper constructs a more efficient and secure chaotic image encryption algorithm than other approaches. Hybrid chaotic mapping and dynamic random growth techniques are used in^45^. These chaotic noises are used to increase the security of the technique and its resistance against classical attacks (e.g. chosen-plaintext).

Non-chaotic encryption techniques (e.g.^46^) use the “manipulate-and-mask” principle to produce the necessary confusion and diffusion for the technique. The manipulation part is ensured by operations such as static substitutions, permutations, and shifting. The mask part depends on the encryption technique and ranges from producing key-based code through performing simple manipulations on the key using patterns such DNA sequences. The techniques^19,31,47–49^ use static substitution and mathematical manipulations to generate sufficient confusion that boosts the security of the ciphertext. The use of the key is restricted to generating a sequence of key-based symbols that are XORed with the ciphertext symbols. The generation process for the key-based symbols depends also on static substitution, symbol swap, and shift operations. DNA-based techniques make use of the sophisticated structures of the DNA sequences of living beings^50–52^. These techniques first manipulate their input using manipulation operations and then hide the resulting messages within the complicated human genomic DNA. As such, the security of such methods is built only partially on the manipulation operations but mainly on the complexity of the DNA. The proposed technique^53^ is based on chaotic maps along with DNA coding. This method has two powerful operations that increase its effectiveness: the confusion of the pixels by transforming the nucleotide into its base pair for random times and the generation of the new keys according to the plain image and the common keys. Authors^54^ proposed a novel image encryption scheme based on DNA sequence operations and a spatiotemporal chaos system to encrypt images.

Authors also proposed many important neural network based encryption techniques. Authors^55^ proposed a double image encryption algorithm based on convolutional neural network and dynamic adaptive diffusion. The technique ensures the security of double image and improves the encryption efficiency and reduces the possibility of being attacked. The technique proposed in^56^ uses continuous-variable quantum neural network to induce high confusion and thus secure the ciphered images.

Honey encryption techniques^57–59^ are designed to withstand the brute-force attacks. The idea is to respond intelligently to incorrect key attempts with a decryption that yields a plausible, but fake document. This way attackers will be confused and have no clue whether they actually getting the right plaintext.

The proposed technique substantially differs from these techniques and has better security. Although the “manipulate-and-mask” techniques show reasonably high diffusion and confusion, they lack the capabilities of the proposed method. First, the proposed method utilizes a chaotic-driven scheme that makes the substitution and replacement inherently chaotic. This is significantly more effective than a pure static substitution and deterministic manipulation operations. The DNA techniques capitalize on the complexity of the DNA sequences to secure the information. The proposed technique, however, has a robust computational model that produces complicated hiding code that resembles the DNA sequences. The hiding code production is based on the encryption key, which fits perfectly with the main objective of encryption “the encryption security is fully built on the key”. The Honey techniques base their security on the ability to confuse attackers. The proposed technique uses better techniques that significantly raise the confusion due to chaos driven behavior. Additionally, the proposed technique is more general. The Honey techniques are applicable only on short plaintext.

## Conclusions and future work

The paper presented an encryption technique. The encryption technique includes the space-shifting operations, which uses chaotic behavior (e.g. chaotic neural network and chaotic substitution) to greatly eliminate structural relation to the plaintext. The technique also includes a sealing layer, which uses principles from the color theory and chaotic systems to produce highly complicated codes for forcing further confusion in the ciphertext. The performance of the technique is thoroughly tested using data sets that consider the key/plaintext variations and entropy. The randomness tests (NIST and ENT batteries) showed high performance. Large ratio of the sequences passed the randomness tests.

The proposed technique has several merits. First, the space chaotic shift process uses three principal subprocesses. Each subprocess has a nonlinear functionality that makes drastic changes to its input. The chaotic neural network substitutes the input symbols by mixing these symbols with chaotic layers using additions and multiplication in Galois field $GF(2^p)$. The chaotic substitution substitutes the input symbols by influencing them using data-dependent chaotically generated values. Each input symbol is influenced by handling it along with the chaotic value using an *XOR* operation. The chaotic mutation imposes micro changes (bit-level) changes to the symbol using chaotic operation. The collective impact of the thee subprocesses results in a large confusion to the output. Second, the encryption technique is lightweight (time-wise) and can produce maximum confusion using only four rounds. For instance, the sealing layer needed only four rounds to produce a code sequence that passed tests of entropy, avalanche, and randomness (please see Figs. 17, 18, and Table 3). Third, both the chaotic system parameters initializer and the sealing code layer conservatively use the encryption key to effectively hide its identity. The chaotic system parameter initializer applies partial splitting, randomization, and shift-XOR operation to process the key. The sealing layer uses the diffusion stage and other color theory-based operations to avoid having any trace of the key in the resulting sealing code.

We have two directions for future work. We plan to use more test cases. We also plan to use our technique in image encryption. We think that this method is likely to outperform the current image encryption techniques because it has a powerful ability to induce confusion and high bit-distortion capabilities.

## Data Availability

All data generated or analysed during this study are included in this published article.
